# SigB Is a Dominant Regulator of Virulence in *Staphylococcus aureus* Small-Colony Variants

**DOI:** 10.1371/journal.pone.0065018

**Published:** 2013-05-21

**Authors:** Gabriel Mitchell, Alexandre Fugère, Karine Pépin Gaudreau, Eric Brouillette, Eric H. Frost, André M. Cantin, François Malouin

**Affiliations:** 1 Centre d'étude et de valorisation de la diversité microbienne, Département de biologie, Faculté des sciences, Université de Sherbrooke, Sherbrooke, Quebec, Canada; 2 Département de microbiologie et d'infectiologie, Faculté de médecine et des sciences de la santé, Université de Sherbrooke, Sherbooke, Quebec, Canada; 3 Unité de recherche pulmonaire, Faculté de médecine et des sciences de la santé, Université de Sherbrooke, Sherbrooke, Quebec, Canada; University Hospital Münster, Germany

## Abstract

*Staphylococcus aureus* small-colony variants (SCVs) are persistent pathogenic bacteria characterized by slow growth and, for many of these strains, an increased ability to form biofilms and to persist within host cells. The virulence-associated gene expression profile of SCVs clearly differs from that of prototypical strains and is often influenced by SigB rather than by the *agr* system. One objective of this work was to confirm the role of SigB in the control of the expression of virulence factors involved in biofilm formation and intracellular persistence of SCVs. This study shows that extracellular proteins are involved in the formation of biofilm by three SCV strains, which, additionally, have a low biofilm-dispersing activity. It was determined that SigB activity modulates biofilm formation by strain SCV CF07-S and is dominant over that of the *agr* system without being solely responsible for the repression of proteolytic activity. On the other hand, the expression of *fnbA* and the control of nuclease activity contributed to the SigB-dependent formation of biofilm of this SCV strain. SigB was also required for the replication of CF07-S within epithelial cells and may be involved in the colonization of lungs by SCVs in a mouse infection model. This study methodically investigated SigB activity and associated mechanisms in the various aspects of SCV pathogenesis. Results confirm that SigB activity importantly influences the production of virulence factors, biofilm formation and intracellular persistence for some clinical SCV strains.

## Introduction

*Staphylococcus aureus* is a widespread Gram positive pathogen that causes serious problems in health care settings and in the community because of antibiotic resistance and high morbidity and mortality rates [1], [2]. This bacterium has the ability to cause both acute and chronic infections in several organs and expresses numerous virulence factors that are involved in a wide range of pathogenic processes [3]. It is thought that particular sets of virulence factors are required for specific steps during disease progression or for infection of specific organs.

The expression of *S. aureus* virulence factors is controlled by complex regulatory networks and as a function of the bacterial population density and environment. During *in vitro* growth, the transition from the expression of adhesins and other cell-surface proteins to that of exoproteins (hemolysins, proteases and nucleases) involves the activation of the quorum-sensing *agr* system that influences virulence gene expression and mainly depends on a regulatory RNA, RNAIII [4], [5]. Although the functionality of the *agr* system has been reported to be important for the pathogenesis of *S. aureus* in several experimental infection models, it is possible that low *agr* activity could be advantageous during specific diseases [4] such as chronic pulmonary infections in CF patients [6].

Several *S. aureus* components are involved in the regulation of virulence factors including two-component signal transduction systems and regulatory factors, which allow the bacterium to adjust its genetic expression program as a function of its surrounding [3]. The alternative transcription factor sigma B (SigB) is known to affect the expression of several genes encoding virulence factors and stress-response proteins, and seems to counterbalance the influence of the *agr* system on the expression of virulence factors. The activity of SigB peaks early during the stationary phase of growth [7] and some genes (*e.g. asp23*) are expressed as a direct function of SigB activity. SigB is also known to support the expression of some genes in early exponential growth (*e.g. fnbA*) while having a negative effect on the expression of other genes encoding exoproteins (*e.g. hla, sspA, nuc1*) in late exponential growth [8]. Importantly, many genes regulated by SigB are not preceded by a SigB promoter and the effect of SigB on the expression of those genes is likely to be mediated by downstream regulators [8]. The *agr* system and SigB are known to be interconnected [9].

Biofilms are microbial communities embedded in a matrix of extracellular polymeric substances (EPS) that can adhere to either biological or non-biological substrates. Importantly, biofilms are deemed to be involved in persistent infections. The formation of a biofilm is initiated by the adhesion of bacteria to a substrate and involves a maturation phase characterized by intercellular aggregation and the acquisition of an architecturally complex tridimensional structure. Several mechanisms of biofilm formation exist in *S. aureus* [10]. While some staphylococcal strains use an *ica*-dependent polysaccharide matrix, the ability to form biofilms seems to involve *ica*-independent mechanisms in several other strains [11]–[13]. In many cases, intercellular aggregation has been shown to be mediated by cell-surface proteins such as protein A, biofilm-associated protein (Bap) or FnBPs [10]. Extracellular DNA (eDNA) also seems to be an important EPS in many bacterial biofilms and *S. aureus* possesses mechanisms to incorporate DNA into biofilm matrixes [14]–[16]. On the other hand, the dispersal of biofilms involves mechanisms that allow the detachment of bacterial cells or biofilm fragments and the dissemination of the pathogen in the environment or the host [10], [17]. The *agr* system and SigB are among the numerous regulators of virulence known to modulate biofilm formation and dispersion by controlling the expression of both adhesion and dispersion factors (*e.g.* exoproteases and nucleases) [10].

*S. aureus* is now considered a facultative intracellular pathogen. Although the precise role of intracellular infections is still an object of debate for staphylococci, it has been speculated that the intracellular persistence of bacteria may confer protection against the host immune system and extracellular antibiotics [18], [19]. The best characterized mechanism of cell invasion used by *S. aureus* involves the formation of a fibronectin bridge between bacterial FnBPs and the host α_5_β_1_ integrin, but FnBPs-independent mechanisms also exist [19]. Once *S. aureus* is within a host cell, several scenarios were reported to occur and it is likely that a wide range of different outcomes are possible according to bacterial strains and cell types [19]. Nevertheless, it has been demonstrated that the *agr* system is required to escape a phagolysosome [20], [21] and that both the *agr* system and SigB influence the induction of host cell death [22], [23].

Small-colony variants (SCVs) constitute a subpopulation of oxidative phosphorylation-deficient bacteria that grow slowly and differ from prototypical strains in various aspects. SCVs usually are less pigmented, less susceptible to aminoglycoside antibiotics, have altered biochemical properties and low hemolytic activity [18], [24]. Importantly, SCVs are usually recovered from several different types of persistent infections [24] such as those found in the lungs of cystic fibrosis (CF) patients [24], [25]. In experimental infection models, SCVs have the ability to cause infections and to persist although they appear less virulent [24]. The appearance and role of SCVs during infections are still unclear but some results suggest that this phenotype is more difficult to eradicate with antibiotic treatment *in vivo* [26], [27]. An outstanding study has recently demonstrated that SCVs emerge during long-term infections and are associated with a weaker immune response [28]. The contribution of specific virulence factors in the establishment of SCV infections remains an incompletely investigated research field.

The production of virulence factors is altered in SCVs [25], [29], [30]. The expression profile of virulence genes in some SCVs has been attributed to an atypical activity of global regulators where SigB activity is constitutive and that of the *agr* system is low [25], [29]. More precisely, SigB and *agr* activities seem to explain the constitutive expression of some cell-surface proteins and the down-regulation of several exoproteins in those SCVs [31], [32]. However, the relative roles of SigB and *agr* that may explain some of the characteristics of SCVs are still speculative and are only based on interpretations of gene expression profiles. Interestingly, some SCV strains produce relatively higher amounts of biofilms than prototypical strains [32], [33] and persist more efficiently inside non-phagocytic host cells [34], which both, alone or together, may explain the association of these bacteria with chronic infections. Despite that, molecular mechanisms involved in the ability of SCVs to form biofilms and to persist within host cells have not been thoroughly investigated although it was shown that SigB may contribute to these phenotypes in SCVs [25], [32]. The aim of this study was to investigate the role of SigB-dependent mechanisms in the various aspects of the pathogenesis of SCVs. Our results underline the major role played by SigB in the pathogenesis of SCVs through its influence on virulence factors production, biofilm formation and intracellular persistence.

## Results

### Formation of biofilms by SCVs may involve extracellular proteins

**Figure 1:**
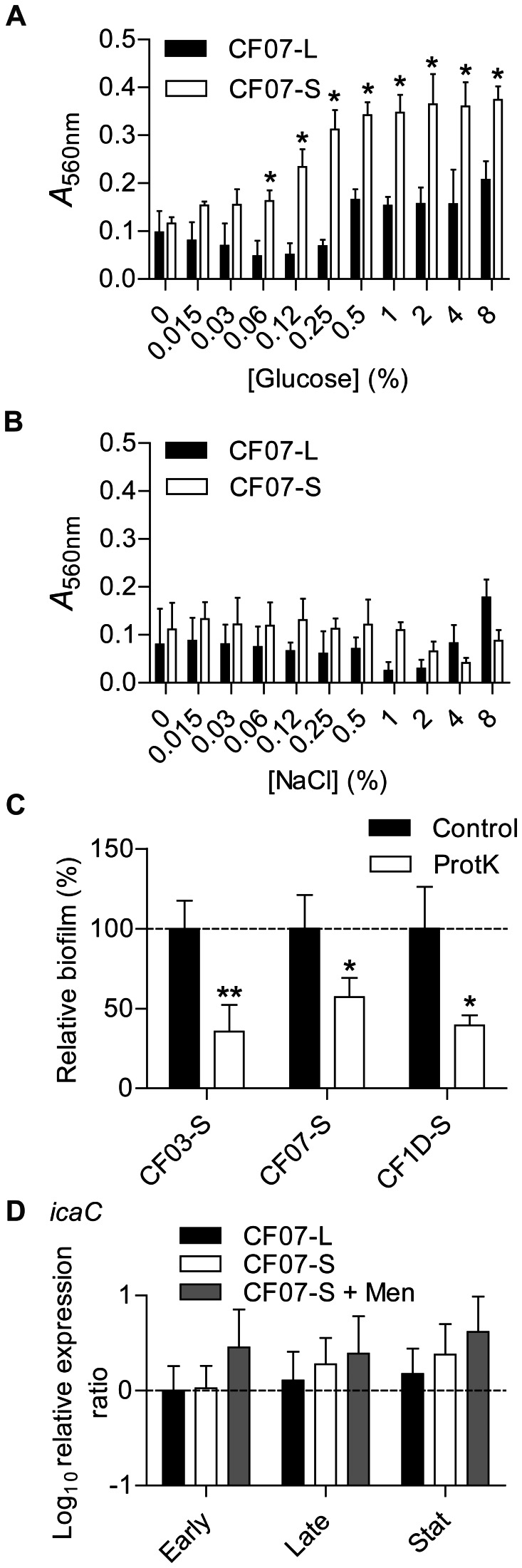
Formation of biofilms by SCVs may involve extracellular proteins. The extent of biofilm production was measured by crystal violet staining (A_560 nm_) of normal CF07-L and SCV CF07-S strains as a function of glucose (A) and NaCl (B) concentrations following a 48-h incubation period. Statistically significant differences between strains are indicated for each concentration of glucose and NaCl (*P*<0.05; two-way ANOVA with Bonferonni's posttest, *n* = 3). (C) Susceptibility of SCVs' biofilms to treatment with proteinase K. Statistically significant differences between control and treated conditions are indicated (*, *P*<0.05; **, *P*<0.01; unpaired *t* test, *n* = 3). Results are normalized according to the control condition for each strain. (D) Expression ratio of the *icaC* gene as a function of growth for strains CF07-L, CF07-S and CF07-S in the presence of menadione, which restores normal growth. Results are expressed according to CF07-L in the early exponential phase of growth. No statistically significant difference was revealed between conditions for each growth phase (ANOVA with Dunnett's posttest, *n* = 3–4). Results are expressed as means with standard deviations.

Mechanisms involved in biofilm production by SCVs were first investigated in presence of glucose and NaCl and by comparing biofilm formation for the genetically-related CF07-L (prototypical) and CF07-S (SCV) strains co-isolated from a CF patient. Glucose and NaCl are known to stimulate the production of protein- and polysaccharide-dependent biofilms in *S. aureus*, respectively [11]. Figure 1A and 1B show that the SCV CF07-S strain formed more biofilm than the normal CF07-L strain in the presence of increasing amounts of glucose (as shown previously [32], [33]), but not in the presence of NaCl. Detachment assays using proteinase K confirmed that extracellular proteins are involved in biofilms produced by three different SCV isolates from CF (Figure 1C). SCVs CF03-S and CF1D-S were already shown to produce greater amounts of biofilm than their normal co-isolated counterparts [33] and were here used to confirm results obtained with strain CF07-S. In order to support the hypothesis that polysaccharides are not involved in the greater biofilm formation ability of CF07-S, qPCR was performed to compare the expression of *icaC* in CF07-L and CF07-S from planktonic cultures and no statistically significant difference was accordingly observed (Figure 1D). Menadione was also used to supplement and restore normal growth levels for CF07-S during experiments measuring the expression of *icaC* at different phases of growth. It was previously shown that menadione abolished the production of biofilm by CF07-S [32]. Figure 1D confirmed that restoring the oxidative phosphorylation and the metabolic status of CF07-S by menadione supplementation does not alter the expression of *icaC* (*P*>0.05). This section supports the hypothesis that extracellular proteins are involved in the formation of biofilms by the three SCV strains tested.

### SCVs may have low biofilm-dispersing proteolytic activity

**Figure 2:**
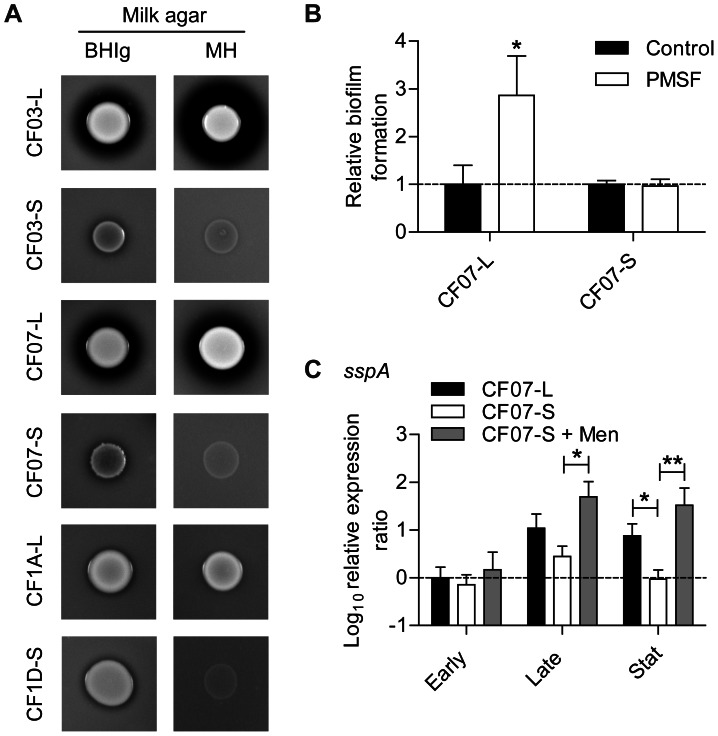
SCVs may have low biofilm-dispersing proteolytic activity. (A) The proteolytic activities of normal (CF03-L, CF07-L and CF1A-L) and SCV (CF03-S, CF07-S and CF1D-S) strains were evaluated on BHI supplemented with glucose (BHIg) or MH milk-agar after 24 and 48 h, respectively. (B) Relative biofilm formation of CF07-L and CF07-S in the presence of the serine-protease inhibitor PMSF following 48 h of incubation. Results are normalized according to the control condition for each strain. A statistically significant difference is indicated between control and treated CF07-L (*, *P*<0.05; unpaired *t* test, *n* = 3). (C) Expression ratio of the *sspA* gene as a function of growth for strains CF07-L, CF07-S and CF07-S in the presence of menadione. Results are expressed according to CF07-L in the early exponential phase of growth. Statistically significant differences to CF07-S are indicated for each growth phase (*, *P*<0.05; **, *P*<0.01; ANOVA with Dunnett's posttest, *n* = 3–4). Results are expressed as means with standard deviations.

It is known that proteinaceous biofilms are dispersed by mechanisms involving extracellular proteases [17]. Experiments were thus performed to evaluate the proteolytic activity of SCVs in comparison to their normal counterparts and demonstrated that SCVs have no or low proteolytic activity (Figure 2A). Two types of plates supplemented with non-fat dry milk (BHI glucose and MH agar) were used to confirm that results are reproducible in different media. It is noteworthy that the low (or null) proteolytic activity of SCVs was still observed on either media even after 48 hours of incubation (Figure 2A and data not shown). Also, the presence of the serine protease inhibitor PMSF increased biofilm production for strain CF07-L, but not for the already high producer CF07-S (Figure 2B), thus confirming the association between proteolytic activity and biofilm dispersion in the CF07 strain background. Accordingly, the expression of the V8 serine protease gene (*sspA*) was down-regulated in CF07-S in comparison to the prototypical CF07-L strain in stationary phases of growth (Figure 2C). No statistically significant difference was detected between CF07-L and CF07-S in the expression of the protease gene *aur* (Figure S1). Moreover, the transcripts for both *splA* and *splC* were not detected in the CF07 background (data not shown), which suggests that serine protease-like proteins from the *spl* operon are not contributing to biofilm dispersion in these strains. These results suggest that the proteolytic dispersion of biofilm is not an active process in these SCVs.

### SigB modulates biofilm formation in SCV CF07-S but is not solely responsible for the repression of proteolytic activity

**Figure 3:**
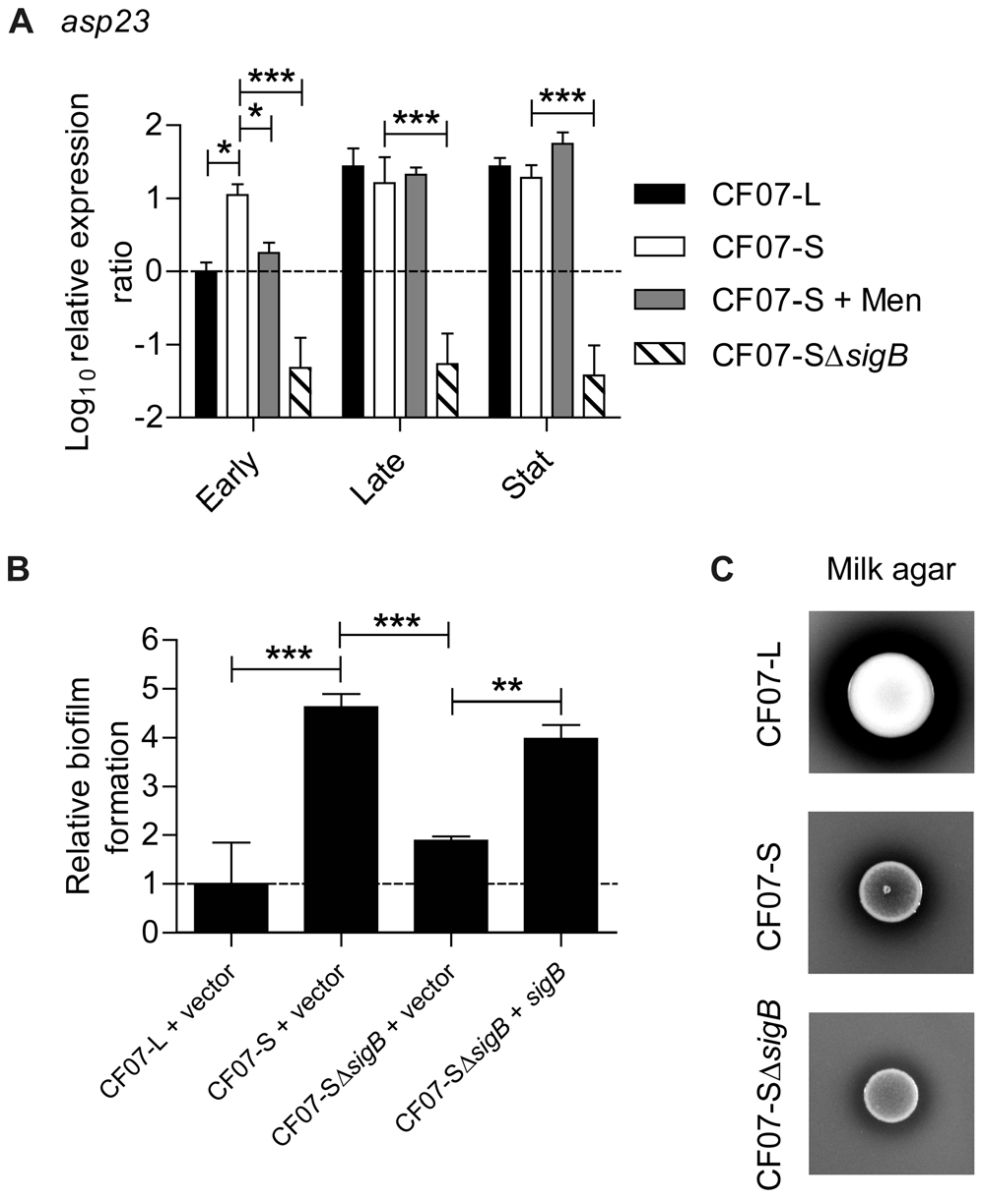
SigB modulates biofilm formation in SCV CF07-S but is not solely responsible for the repression of proteolysis. (A) Expression ratio of the *asp23* gene as a function of growth for strains CF07-L, CF07-S, CF07-S in the presence of menadione and CF07-SΔ*sigB*. Results are expressed according to CF07-L in the early exponential phase of growth. Statistically significant differences relative to CF07-S are indicated for each growth phase (*, *P*<0.05; ***, *P*<0.001; ANOVA with Dunnett's posttest, *n* = 3–4). (B) Relative biofilm formation of CF07-L, CF07-S and CF07-SΔ*sigB* carrying the empty vector (pFM1) or the *sigB* expression vector (pFM2) following 48 h of incubation in the presence of 0.25 µM CdCl_2_. Statistically significant differences are indicated (**, *P*<0.01; ***, *P*<0.001; ANOVA with Tuckey's posttest, *n* = 3). Results are expressed as means with standard deviations. (C) Proteolytic activity of CF07-L, CF07-S and CF07-SΔ*sigB* on BHIg milk-agar following 48 h of incubation.

We previously showed that SigB affects the biofilm formation of CF07-S [32]. The activity of SigB was monitored using qPCR targeting *asp23*, a known marker of SigB activity [35]. Figure 3A confirmed that the expression of *asp23* is higher in SCV CF07-S in comparison to the prototypical strain CF07-L in the early exponential growth (*P*<0.05, ANOVA with Dunnett's posttest). This confirms that a high SigB activity is seen throughout the growth phases in SCV CF07-S because of its metabolic status (*i.e.* electron transport deficiency), whereas the expression of the SigB marker *asp23* only increases toward the stationary phase in normal strains. Noteworthy, the SCV strains CF03-S and CF1D-S are also characterized by a higher SigB activity in comparison to normal-growing *S. aureus* bacteria [25] (data not shown). Figure 3B confirms that the formation of biofilm by CF07-S is indeed regulated by SigB by using a *sigB* mutant strain carrying an empty vector (pFM1) or a *sigB* expression vector (pFM2). It has been demonstrated that SigB modulates the dispersion of proteinaceous biofilm [36], [37] as well as the expression of exoproteases such as Aur and SspA in normal strains [36]. In contrast to that, Figure 3C shows that the proteolytic activity of SCV CF07-SΔ*sigB* remains low and is not altered in comparison to SCV CF07-S. However, the expression of *sspA* in CF07-SΔ*sigB* was increased in comparison to that of CF07-S in the stationary phase of growth (Figure S2). In order to confirm that the activity of serine-proteases (such as SspA) was not involved in the dispersal of biofilm from CF07-SΔ*sigB*, we performed biofilm formation with CF07-SΔ*sigB* exposed to PMSF and did not observe any statistically differences in comparison to the untreated control (Figure S3). Our results thus support the idea that serine-proteases are not involved in the dispersion of the biofilm forms by CF07-SΔ*sigB*, although a higher transcription of the *sspA* gene can be detected in this strain in comparison to CF07-S. Interestingly, the proteolytic activity was modulated in a SigB-dependent manner when CF07-S was exposed to menadione (Figure S4). Overall, this section suggests that the regulation of biofilm formation by SigB does not involve proteolytic mechanisms in the SCV CF07-S.

### The regulation of the agr system by SigB influences the hemolytic activity and the formation of biofilm in SCV CF07-S

**Figure 4:**
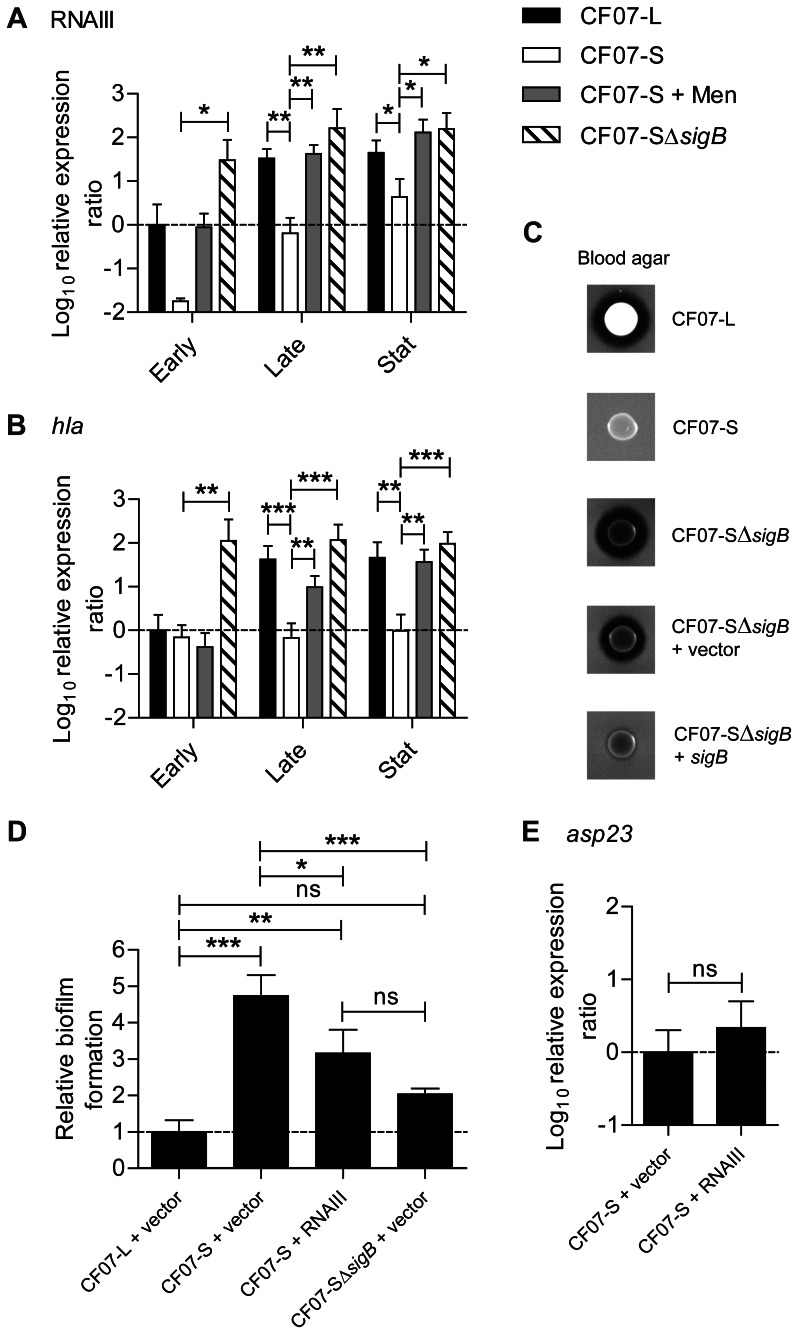
The *agr* system is influenced by SigB and modulates hemolysis and biofilm formation in SCV CF07-S. Expression ratio of RNAIII (A) and the *hla* gene (B) as a function of growth for strains CF07-L, CF07-S, CF07-S in the presence of menadione and CF07-SΔ*sigB*. Results are expressed according to CF07-L in the early exponential phase of growth. Statistically significant differences to CF07-S are indicated for each growth phase (*, *P*<0.05; **, *P*<0.01; ***, *P*<0.001; ANOVA with Dunnett's posttest, *n* = 3–6). (C) Hemolytic activity of CF07-L, CF07-S, CF07-SΔ*sigB* and CF07-SΔ*sigB* carrying the empty vector (pFM1) or the *sigB* expression vector (pFM2) following 48 h of incubation on blood-agar plates supplemented with 0.25 µM CdCl_2_. (D) Relative biofilm formation of CF07-L, CF07-S and CF07-SΔ*sigB* carrying the empty vector (pFM1) or the RNAIII expression vector (pFM4) following 48 h of incubation in the presence of 0.12 µM CdCl_2_. Statistically significant differences are indicated (ns, non statistically significant; *, *P*<0.05; **, *P*<0.01; ***, *P*<0.001; ANOVA with Tuckey's posttest, *n* = 3). (E) Expression ratio of the *asp23* gene for CF07-S carrying the empty vector (pFM1) or the RNAIII expression vector (pFM4) grown to mid-exponential phase in the presence of 0.12 µM CdCl_2_. No statistically significant difference was revealed by an unpaired *t* test (*n* = 3). Results are expressed as means with standard deviations.

The activity of the *agr* system is influenced by SigB [9], [32] and is repressed in SCVs [25], [29]. Figure 4A confirms that the expression of RNAIII (the effector of the *agr* system) is repressed by SigB in the SCV CF07-S. Accordingly, the inactivation of *sigB* strongly up-regulated the α-hemolysin (*hla*) gene (Figure 4B), which expression is already known to be influenced by *agr* activity [3]. Moreover, the inactivation of *sigB* in this SCV strain triggers an increase in hemolytic activity (Figure 4C), which is overall controlled by the *agr* system, although not solely through Hla expression [38], [39]. In order to know whether the control of the *agr* system by SigB is involved in the formation of biofilm by CF07-S, a RNAIII expression vector was introduced in this strain (pFM4), which was expressing over 10-fold more RNAIII transcript than the empty vector control strain when in presence of the transcriptional inducer (data not shown). Figure 4D shows that the overexpression of RNAIII reduced the formation of biofilm by CF07-S (*P*<0.05, ANOVA with Tuckey's posttest), but not to a level as low as seen with CF07-L. The level of expression of *asp23* was evaluated in CF07-S over-expressing RNAIII and it showed that RNAIII does not modulate SigB activity in this SCV. Accordingly, exposure of a SCV strain to overnight culture supernatants from a normal strain with the same *agr* type did not influence the expression level of *asp23* (data not shown). Overall, this section confirms that the influence of SigB on the *agr* system is unidirectional and influences both hemolysis and biofilm formation in SCVs.

### The SigB-dependent expression of fnbA contributes to biofilm formation in SCV CF07-S

**Figure 5:**
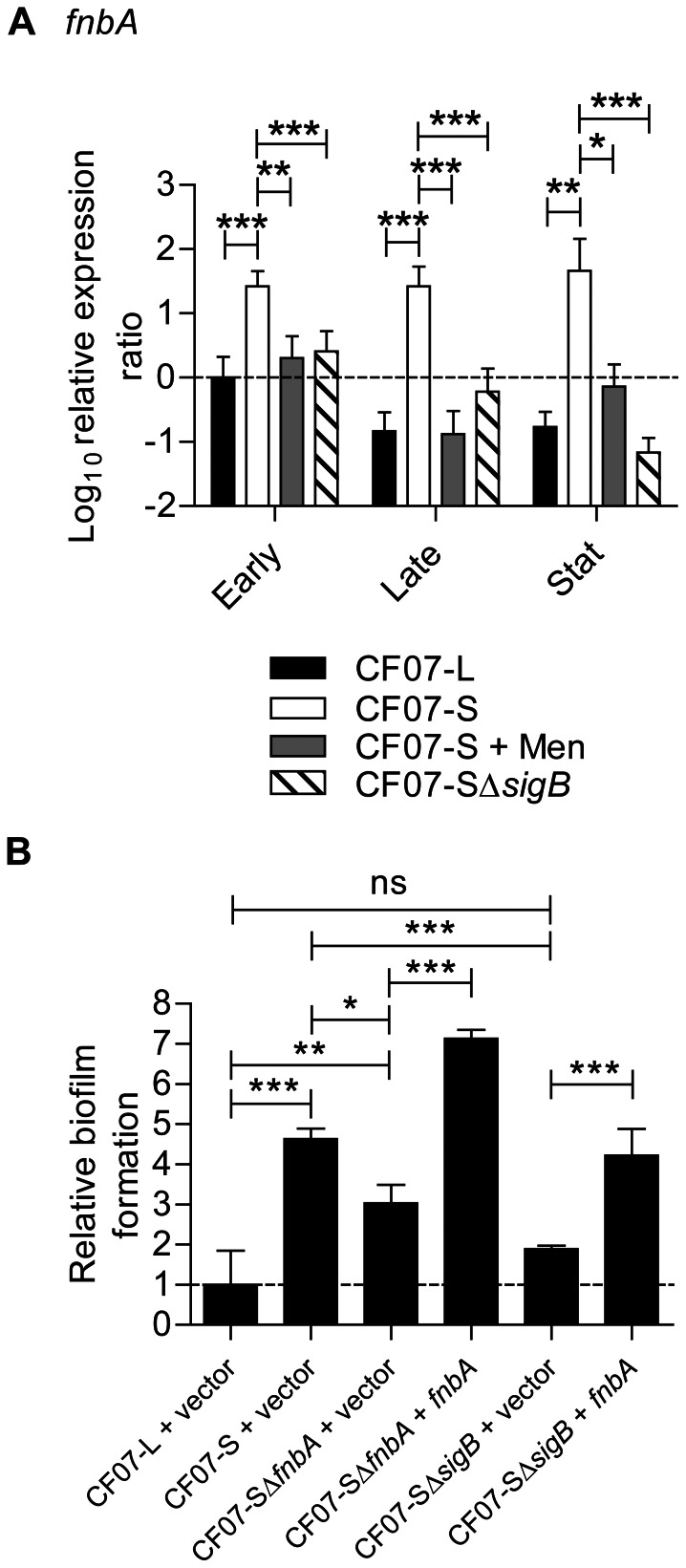
The SigB-dependent expression of FnBPA contributes to biofilm formation in SCV CF07-S. (A) Expression ratio of the *fnbA* gene as a function of growth for strains CF07-L, CF07-S, CF07-S in the presence of menadione and CF07-SΔ*sigB*. Results are expressed according to CF07-L in the early exponential phase of growth. Statistically significant differences to CF07-S are indicated for each growth phase (*, *P*<0.05; **, *P*<0.01; ***, *P*<0.001; ANOVA with Dunnett's posttest, *n* = 4–9). (B) Relative biofilm formation of CF07-L, CF07-S, CF07-SΔ*fnbA* and CF07-SΔ*sigB* carrying the empty vector (pFM1) or the *fnbA* expression vector (pFM3) following 48 h of incubation in the presence of 0.25 µM CdCl_2_. Relevant statistically significant differences are indicated (ns, non statistically significant; *, *P*<0.05; **, *P*<0.01; ***, *P*<0.001; ANOVA with Tuckey's posttest, *n* = 3). Results are expressed as means with standard deviations.

It has been shown that FNBPs are involved in the formation of proteinaceous biofilms in normal *S. aureus* strains [12] and that some SCVs strongly expressed *fnbA* throughout the growth phases because of a sustained SigB activity [31]. Figure 5A confirms that CF07-S strongly expressed *fnbA* in a SigB-dependent manner throughout growth in comparison to normal bacteria. The inactivation of *fnbA* decreased the biofilm formation of CF07-S (*P*<0.05, ANOVA with Tuckey's posttest), but not to the level of CF07-L, which suggests that some additional *fnbA*-independent mechanisms may also be involved in the biofilm formation of CF07-S (Figure 5B). However, expression of *fnbA* in CF07-SΔ*sigB* restored biofilm formation to the level of CF07-S (Figure 5B). These results suggest that *fnbA* is importantly involved in the biofilm formation of the SCV strain CF07-S.

### Extracellular DNA may be a component of the biofilm formed by SCVs

**Figure 6:**
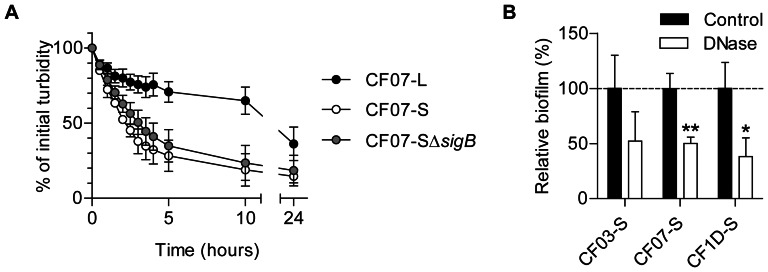
Extracellular DNA may be a component of the biofilm formed by SCVs. (A) Autolysis of the CF07-L, CF07-S and CF07-SΔ*sigB* strains as a function of time. Results are expressed as percentages of the initial turbidity for each condition. (B) Susceptibility of SCVs' biofilms to treatment with DNase I. Statistically significant differences between control and treated conditions are indicated (*, *P*<0.05; **, *P*<0.01; unpaired *t* test, *n* = 3–4). Results are normalized according to control condition for each strain. Results are expressed as means with standard deviations.

Extracellular DNA released during autolysis of bacterial sub-populations is now recognized as an important component of biofilm matrices [14], [16], [17]. We speculated that SigB activity modulates autolysis and the release of extracellular DNA in SCVs. Figure 6A shows that CF07-S bacteria lysed at a rate strikingly higher than CF07-L, but that SigB was not involved in the control of this process. Noteworthy, Triton induced autolysis of strain CF07-L but did not alter that of strains CF07-S and CF07-SΔ*sigB* (Figure S5). The role of extracellular DNA in the formation of biofilm by three SCVs is confirmed on Figure 6B showing the susceptibility of the SCVs' biofilms to treatment with DNase I. Although the release of extracellular DNA by autolysis seemed to be more important in the SCV CF07-S than in its normal counterpart CF07-L and that this DNA release may be involved in the formation of biofilms, this mechanism was not modulated by SigB activity.

### The nuclease activity of SCV CF07-S is repressed through a SigB-dependent mechanism

**Figure 7:**
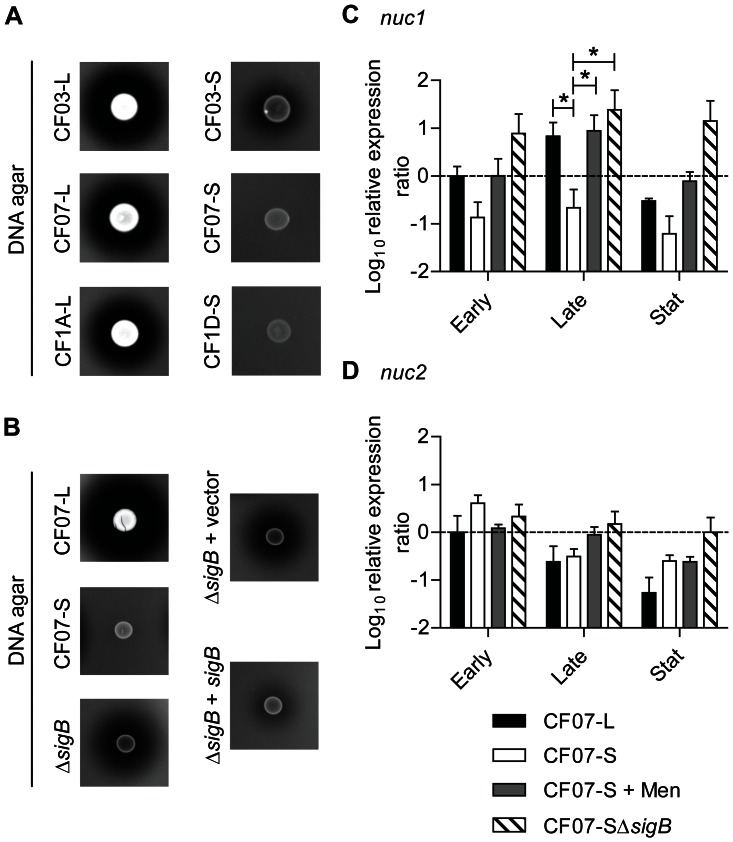
The nuclease activity of SCV CF07-S is repressed through a SigB-dependent mechanism. (A) The nuclease activity of normal (CF03-L, CF07-L and CF1A-L) and SCV (CF03-S, CF07-S and CF1D-S) strains was evaluated on DNA agar following 24 h of incubation. (B) The nuclease activity of CF07-L, CF07-S, CF07-SΔ*sigB* and CF07-SΔ*sigB* carrying the empty vector (pFM1) or the *sigB* expression vector (pFM2) was evaluated on DNA agar following 48 h of incubation in the presence of 0.25 µM CdCl_2_. Expression ratios of *nuc1* (A) and *nuc2* (B) genes as a function of growth for strains CF07-L, CF07-S, CF07-S in the presence of menadione and CF07-SΔ*sigB*. Results are expressed according to CF07-L in the early exponential phase of growth. Statistically significant differences to CF07-S are indicated for each growth phase (*, *P*<0.05; ANOVA with Dunnett's posttest, *n* = 3–4). Results are expressed as means with standard deviations.

Biofilm dispersion was shown to be influenced by a SigB-dependent mechanism controlling nuclease activity in normal *S. aureus* strains [40]. Figure 7A demonstrates that the nuclease activity of each of three SCV strains was lower than that of their normal counterparts. Furthermore, this nuclease activity was controlled by SigB in the SCV strain CF07-S (Figure 7B). Of the two genes encoding nucleases in the *S. aureus* genome (*nuc1* and *nuc2*), only *nuc1* was shown to be modulated by SigB [40]. Accordingly, Figure 7C and 7D demonstrate that *nuc1*, but not *nuc2*, was down-regulated by SigB in the SCV strain CF07-S. This section suggests that the nuclease activity of SCV CF07-S is repressed through the SigB-dependent control of *nuc1* expression.

### SigB is required for the intracellular replication of SCV CF07-S within epithelial cells

**Figure 8:**
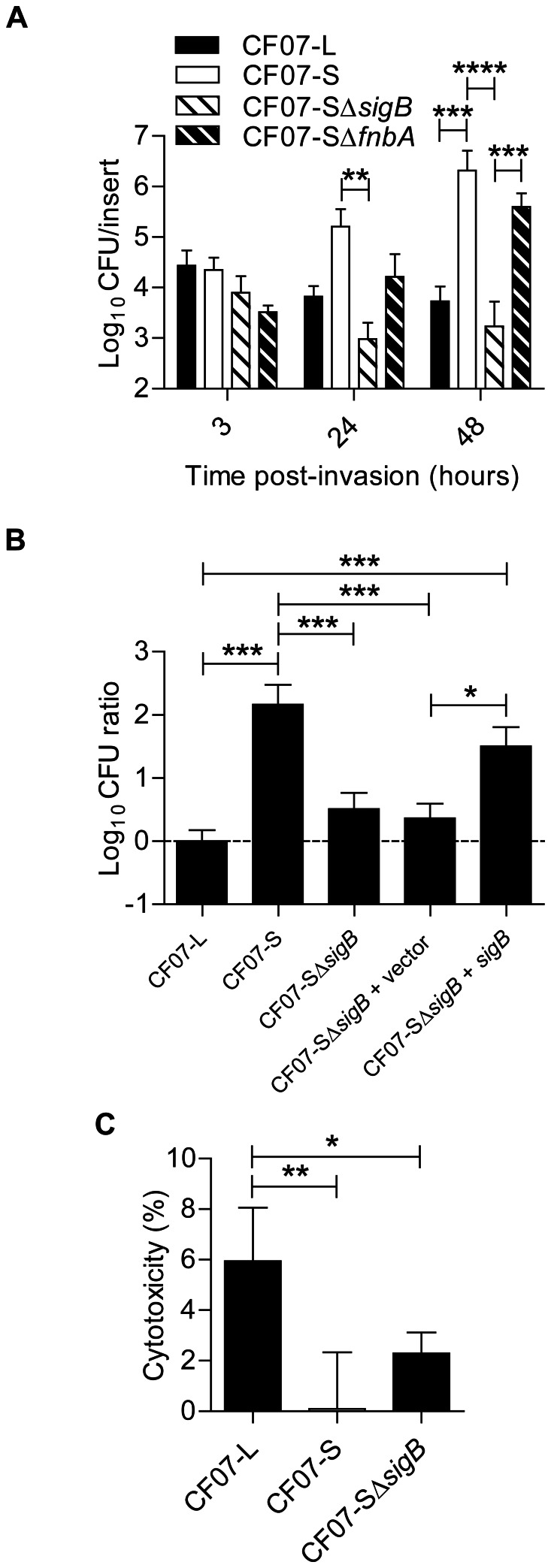
SigB is required for the replication of SCV CF07-S within CF cells. (A) Infection kinetics of shCFTR Calu-3 cells (*i.e.*, CF-like cells) by strains CF07-L, CF07-S, CF07-SΔ*sigB* and CF07-SΔ*fnbA*. Statistically significant differences in CFU/insert recovered from infected cells are indicated (**, *P*<0.01; ***, *P*<0.001 ; ****, *P*<0.0001 ; two-way ANOVA with Bonferonni's posttest, *n* = 3–7). (B) CFU ratios recovered from cells infected with CF07-L, CF07-S, CF07-SΔ*sigB* and CF07-SΔ*sigB* carrying the empty vector (pFM1) or the *sigB* expression vector (pFM2) 48 h post-invasion. For cells infected with strains carrying a vector, the post-invasion media was supplemented with 0.25 µM CdCl_2_. Statistically significant differences between the CFU ratios recovered from cells infected with the different strains are indicated (*, *P*<0.05; ***, *P*<0.001; ANOVA with Tuckey's posttest, *n* = 3–7). (C) Host cell lysis was evaluated 48 h following invasion with CF07-L, CF07-S and CF07-SΔ*sigB* by performing LDH cytotoxicity assays. Statistically significant differences are indicated (*, *P*<0.05; **, *P*<0.01; ANOVA with Tuckey's posttest, *n* = 4). Results are expressed as means with standard deviations.

The role of SigB in the persistence of SCVs within epithelial cells was next studied because it was previously suggested that SigB may contribute to the infection of host cells by SCVs [25]. Figure 8A shows that SCV CF07-S has the ability to accumulate inside CF-like epithelial cells as previously reported [34]. Interestingly, this ability to replicate over time inside CF-like epithelial cells was not observed for the normal CF07-L strain and was dependent on the activity of SigB. Although SigB provided to SCV CF07-S the ability to accumulate intracellularly over time, SigB was not apparently involved in the internalization process as evaluated by our cell infection protocol (Figure 8A, 3 h post-invasion time, *P*>0.05). Initial infection of cells with CF07-SΔ*fnbA* was also not different from that of CF07-S although a trend was observed (Figure 8A, 3 h post-invasion time, *P*>0.05). Many host cell internalization protocols compare bacteria and strains isolated from the exponential phase of growth. Here, we used SCVs collected from a 20-h old agar plate to perform the cell internalization step, a condition where *fnbA* should be strongly up-regulated in CF07-S in comparison to CF07-L (Figure 5A). In such conditions, *fnbA*-dependent differences in internalization between the strains studied here should have been observed if this gene had been by itself an essential determinant in this process. In fact, we showed that a *fnbAB* double mutant in strain 8325-4 was drastically less internalized than its parent strain (Figure S6), which supports the validity of the cell infection protocol used in this study and confirmed the role of at least one or the two FnBPs in internalization of *S. aureus* in polarized CF-like epithelial cells. Moreover, immunolocalization experiments revealed that some α5β1 integrins were at the apical surface of cells (data not shown). The role of SigB in the ability of SCV CF07-S to accumulate within cells was confirmed in Calu-3 and Calu-3 shCFTR_ALTER_ cells, both of which producing a normal CFTR (Figure S7). Figure 8B confirms that SigB was involved in the intracellular accumulation of SCV CF07-S 48 h post-invasion and shows that the expression of *sigB* in CF07-SΔ*sigB* complemented the infection. Interestingly, the extent of epithelial cell death 48 h post-invasion did not follow the intracellular accumulation of SCV CF07-S (Figure 8C). These results suggest that SigB confers on SCV CF07-S the ability to replicate within epithelial cells but that it is not involved in the induction of host cell death.

### SigB provides better fitness to SCV CF07-S in the presence of a normal strain in a mouse pulmonary model of infection

**Figure 9:**
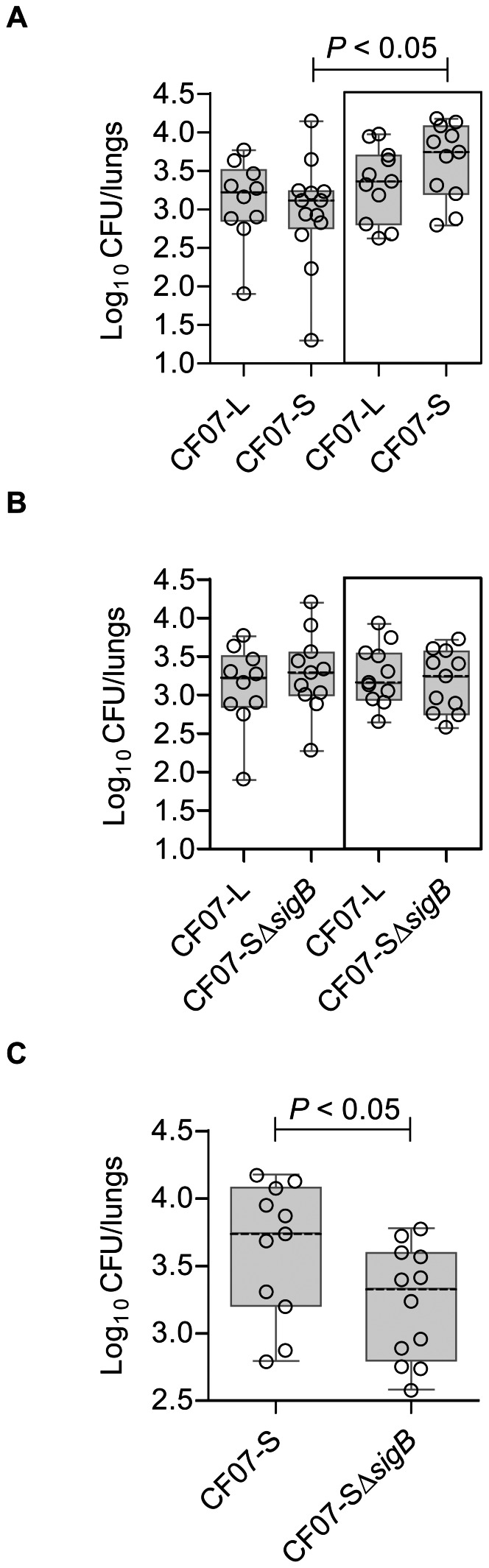
SigB provides better fitness to SCV CF07-S in a mouse pulmonary model of infection. (A) CFU recovered from mouse lungs infected with CF07-L, CF07-S or a combination of both strains (boxed results) 48 h post-inoculation. A statistically significant difference is indicated (*, *P*<0.05; Kruskal-Wallis with a Dunn's posttest, *n* = 10–13). (B) CFU recovered from mouse lungs infected with CF07-L, CF07-SΔ*sigB* or a combination of both strains (boxed results) 48 h post-inoculation. No statistically significant difference was revealed by a Kruskal-Wallis test followed by a Dunn's posttest (*n* = 10–11). (C) CFU recovered from mouse lungs infected with either CF07-S or CF07-SΔ*sigB* during a co-infection with the normal strain CF07-L 48 h post-inoculation. A statistically significant difference is indicated (*, *P*<0.05; Mann-Whitney test, *n* = 11). For a given experiment, the quantity of bacteria in the mixed inoculum was equivalent to the sum of each inoculums prepared for individual strains. The CFU content was evaluated by plating logarithmic dilutions of homogenates on TSA and enumerating normal and SCV colonies.

In order to evaluate if the above-described SigB-dependent phenotypes influence the pathogenesis of SCV infections, a mouse pulmonary infection model was used. We compared pulmonary infections (CFU recovered from the lungs of each mouse) achieved with either the normal strain CF07-L or the SCV strain CF07-S at 48 h post-inoculation in single infections, but also in infections where both normal and SCV strains were combined (*i.e.* a co-infection). Indeed, SCVs are rarely found alone in clinical samples [24], and a co-infection model may be more representative of the clinical setting. Interestingly, Figure 9A shows that more SCVs are recovered from lungs during a co-infection with the normal strain CF07-L than during single infections 48 h post-inoculation. This increase in SCV counts was not observed in lungs infected with CF07-L in combination with CF07-SΔ*sigB* (Figure 9B). Importantly, more SCV counts were recovered from lungs of mice infected with the combination of CF07-L and CF07-S in comparison to that observed with the combination of CF07-L and CF07-SΔ*sigB* (*P*<0.5, Mann-Whitney test) (Figure 9C). This section confirms that SigB may be advantageous to the pathogenesis of SCVs.

## Discussion

Although the role of SCVs in chronic infection is still controversial, more and more studies support the clinical association between these variants and persistent infections. It is possible that the SCV phenotype helps colonization of the host by reducing tissue destruction and activation of the host immune system [24], [28]. However, the fact that some SCVs can form greater amounts of biofilm, that they are able to persist within nonphagocytic host cells and that they can revert to the normal phenotype is worrisome considering that these phenotypes might be involved in chronic infections [18], [41], [42]. This study better defines some of the molecular mechanisms underlying the ability of some SCV strains to form biofilms and to persist within non-phagocytic host cells.

One hypothesis to explain the difference in the expression of virulence genes seen between normal and SCV strains is that the slow growth of SCVs does not allow auto-inducing peptides to reach the extracellular concentration required to activate the *agr* system, which is known to trigger the expression of several virulence factors down-regulated in SCVs [3], [4], [24]. However, results of this study show that SigB activity is dominant over that of the *agr* system in the strain SCV CF07-S. Indeed, whereas the deletion of *sigB* led to overexpression of RNAIII, overexpression of RNAIII did not influence the expression level of *asp23* in this strain. Queck *et al.* [5] demonstrated that, although RNAIII is involved in the regulation of most *agr*-dependent virulence factors, *agr*-dependent genes with metabolic-related functions are regulated through an RNAIII-independent pathway. Hence, it would be relevant to evaluate the effect of *agrA* and *agrACDB* overexpression in the SCV strain CF07-S in order to better study the role of *agr* in the pathogenesis of this SCV. The construction of an *agr*/*sigB* double mutant in the CF07-S background should also help to investigate the interaction between these two regulators. However, results from this study strongly suggest that SigB activation is upstream to any putative *agr* role in the SCV CF07-S. Because several genes of the SigB regulon are thought to be indirectly influenced by SigB [8], future studies should also aim at revealing the role of regulators or regulatory networks activated by SigB in the pathogenesis of SCVs. Interestingly, it was previously shown that the expression of *sarA* is up-regulated through a SigB-dependent mechanism in CF07-S [32].

Some cell-surface protein genes are actively expressed by some SCVs (*e.g. fnbA*, *clfA* and *sceD*) whereas several secreted exoprotein genes are repressed [25]. Interestingly and at least for *fnbA*, this up-regulation is apparently constitutive and observed throughout the growth of some SCVs [31]. In addition to transcriptional and translational regulation, it is known that secreted proteases are used by *S. aureus* to degrade adhesins and to modulate adhesion to host tissues [43]. The low proteolytic activity of SCVs we observed in this study thus supports the hypothesis that the SCV phenotype promotes long-term colonization of host tissues. However, the role of cell-surface proteins (other than FnBPs) in the pathogenesis of SCVs remains to be investigated more thoroughly.

Although the ability of some SCVs to form biofilms is known [32], [33], [44], [45], almost all studies on SCVs focused on their intracellular persistence [18], [24]. Nevertheless, the role of biofilms in chronic infections must not be overlooked, especially in the context of pulmonary infections in CF patients [41], [42]. Until now, mechanisms involved in biofilm formation by SCVs were incompletely understood, although some results suggested that polysaccharide intercellular adhesin (PIA) was a component of their biofilm matrix [44], [45]. However, the roles of SigB, *agr* and FnBPs in biofilm formation of normal strains [10] rather suggested that extracellular proteins may be involved in biofilm formation by SCVs. In the three SCV strains used in this study, we showed that proteins, and to some extent extracellular DNA, are important for biofilm formation and that, at least for strain CF07-S, overexpression of the *ica* operon did not seem to explain the greater production of biofilm in comparison to its normal counterpart CF07-L. However, these results would benefit from a more direct characterization of the components constituting the biofilm produced by CF07-S. Interestingly, it was reported that proteinaceous biofilms are usually devoid of PIA [11]–[13].

Biofilm production is lower in a *sigB* mutant of SCV CF07-S [32]. A role for *fnbA* in the SigB-dependent biofilm formation of this SCV strain is shown by the present study, but a *fnbA* mutation did not completely abolish biofilm formation. As autolysis may be influenced by SigB [46], [47], and because extracellular DNA has been shown to be involved in the formation of a FnBPs/SigB-dependent biofilm [46], it was speculated that a SigB-dependent control of autolysis mediates the release and incorporation of DNA into the biofilm matrix of SCV CF07-S. However, although it was found that the rate of autolysis was drastically higher in the SCV strain CF07-S in comparison to its normal counterpart, SigB did not appear to modulate autolysis in SCVs. Interestingly, it was shown that the proton motive force influences the induction of autolysis [48], which may suggest that the mechanism that controls autolysis in this SCV is upstream to SigB. It was also reported that SigB prevents biofilm dispersion by repressing nuclease activity [40], which is a mechanism we also observed in the SCV CF07-S. Studying the molecular mechanisms controlling extracellular nuclease activity in SCVs should be of interest as SigB was also described to activate *nuc1* expression through the SpoVG-dependent pathway [49]. Overall, SigB positively controls biofilm formation by at least two different mechanisms in the SCV CF07-S: it supports the expression of *fnbA* and represses nuclease activity.

According to this study, the SigB-dependent expression of *fnbA* in SCV CF07-S importantly influences biofilm formation and less so the host cell infection process. However, once the SCV CF07-S is internalized, this study demonstrates that SigB is required for replication and survival within both non-CF and CF-like epithelial cells. It was initially thought that this intracellular accumulation of SCVs was mediated through a SigB-dependent repression of the induction of host cell death, a mechanism that had already been described in other strains [22], [23]. Instead, we observed that while the normal strain CF07-L was more cytotoxic than the SCV strain CF07-S, the deletion of *sigB* did not influence the cytotoxicity of CF07-S in a statistically significant manner. It is thus possible that SigB influences the expression of genes involved in the survival of the SCV CF07-S inside host cells or in the intracellular trafficking of this SCV.

The role(s) of the SCV phenotype during *S. aureus* infections remain to be fully investigated. Moreover, it is not known whether biofilm formation and intracellular persistence are important aspects of the pathogenesis of these strains. The role played by different virulence factors and regulators *in vivo* are even less well known. Indeed, there is no study addressing the importance of specific virulence factors in the pathogenesis of SCVs using experimental models of infections. This study suggests that the colonization of lungs by the SCV CF07-S is helped by co-infection with a normal *S. aureus* strain and perhaps explains why SCVs are rarely recovered alone from clinical specimens [24]. Whether or not tissue damage and inflammation induced by these co-infections are different from that of single infections will be important aspects to investigate in future studies. The *in vivo* dependency of this SCV strain toward the normal phenotype may also explain why the large-scale chromosome flip-flop inversion involved in the reversible switching mechanism of SCV formation recently described for one strain is self-organized to maintain both phenotypes in the bacterial population [50]. This study also supports the hypothesis that SigB is important for the pathogenesis of the SCV CF07-S during lung infection. Additional studies will be necessary to identify the specific SigB-dependent mechanisms involved such as adhesion to host tissues, biofilm formation, intracellular persistence or stress resistance.

Based on the evidence presented here for a central role of SigB in the pathogenesis of SCV CF07-S, therapeutic approaches targeting SigB activity, perhaps in concert with other traditional antibiotics, might offer new alternatives to tackle or prevent chronic infections caused by *S. aureus*. Recently, a small drug-like SigB inhibitor active against *Listeria monocytogenes* and *Bacillus subtilis* was described [51]. Unfortunately, in our hands, we observed no effect for this SigB inhibitor on the expression of *asp23* in the SCV strain CF07-S (data not shown). Nonetheless, screening of compound libraries for inhibitors of *S. aureus* SigB activity might lead to the development of novel therapies interfering with the pathogenesis of SCVs.

## Materials and Methods

### Ethics Statement

The animal experiments were conducted following the guidelines of the Canadian Council on Animal Care and the institutional ethics committee on animal experimentation of the *Faculté des Sciences* of *Université de Sherbrooke*. The institutional ethics committee on animal experimentation of the *Faculté des Sciences* of *Université de Sherbrooke* specifically approved this study (protocol FM2009-01).

### Bacterial strains, cell lines and growth conditions

Strains used in this study are listed in Table 1. CF03-L/CF03-S, CF07-L/CF07-S and CF1A-L/CF1D-S are related pairs of strains co-isolated from CF patients, which respectively have a normal (-L, for large colony) and a SCV (-S, for small colony) phenotype. The genetic relatedness of each strain among pairs was confirmed by the analysis of multiple loci with a variable number of tandem repeats (VNTR) [33]. Multilocus sequence typing (MLST) was also previously performed for strains CF07-L and CF07-S [34]. Except where otherwise stated, *S. aureus* strains were grown in brain heart infusion (BHI) broth (BD, ON, Canada). Whenever required, chloramphenicol (10 µg/ml) (ICN Biomedicals, Irvine, CA), erythromycin (10 µg/ml) (Sigma, Oakville, Ontario, Canada) and tetracycline (10 µg/ml) (Sigma) were added to the media. CdCl_2_ (Sigma) was used to induce the expression of genes under the control of the P*_cad_-cadC* promoter [52]. CdCl_2_ was usually used at a wide range of concentrations for each of the assays performed in this study although only results obtained with the optimal concentration of CdCl_2_ are shown in figures.

**Table 1:** Bacterial strains and plasmids.

Strain or plasmid	Relevant characteristics^a^	Source or reference
***E. coli***		
DH5-α	Host for DNA cloning	-
***S. aureus***		
RN4220	Restriction-defective	-
CF03-S	SCV strain isolated from a CF patient	[25]
CF03-L	Normal strain co-isolated with CF03-S	[33]
CF07-S	SCV strain isolated from a CF patient	[25]
CF07-L	Normal strain co-isolated with CF07-S	[33]
CF1D-S	SCV strain isolated from a CF patient	[33]
CF1A-L	Normal strain co-isolated with CF1D-S	[33]
CF07-SΔ*sigB*	CF07-S Δ*sigB*::*ermA*; Em^r^	[31]
CF07-SΔ*fnbA*	CF07-S Δ*fnbA*::*ermA*; Em^r^	This study
FM1	CF07-S Δ*sigB* (pFM1), Em^r^, Tc^r^	This study
FM2	CF07-S Δ*sigB* (pFM2), Em^r^, Tc^r^	This study
FM3	CF07-S Δ*sigB* (pFM3), Em^r^, Tc^r^	This study
FM4	CF07-S Δ*fnbA* (pFM1), Em^r^, Tc^r^	This study
FM5	CF07-S Δ*fnbA* (pFM3), Em^r^, Tc^r^	This study
FM6	CF07-S (pFM1), Em^r^, Tc^r^	This study
FM7	CF07-S (pFM4), Em^r^, Tc^r^	This study
**Plasmids**		
pBT-2	*E. coli*-*S. aureus* shuttle vector, Ts, Ap^r^, Cm^r^	[54]
pBT-E	*ermA* inserted in pBT-2, Ts, Ap^r^, Cm^r^, Em^r^	[31]
pBT2-Δ*fnbA*::*ermA*	pBT-E with the mutant allele for *fnbA* deletion, Ts, Ap^r^, Cm^r^, Em^r^	This study
pCN36	*S. aureus-E. coli* shuttle vector, pT181-*cop-wt repC*, Ap^r^, Tc^r^	[52]
pCN44	*S. aureus-E. coli* shuttle vector, P*_cad_*-*cadC*	[52]
pFM1	P*_cad_*-*cadC* inserted in pCN36, Tc^r^	This study
pFM2	*sigB* inserted in pFM1, Tc^r^	This study
pFM3	*fnbA* inserted in pFM1, Tc^r^	This study
pFM4	RNAIII inserted in pFM1, Tc^r^	This study

a Ts, *S. aureus* temperature-sensitive replicon; Ap^r^, ampicillin resistant (*E. coli*); Cm^r^, chloramphenicol resistant; Em^r^, erythromycin resistant; Tc^r^, tetracycline resistant.

The human airway epithelial Calu-3 cell line (ATCC HTB 55) and its derivatives (shCFTR_ALTER_ and shCFTR) [53] were cultured at the air-liquid interface as previously described [34]. Cell culture reagents were purchased from Wisent (St-Bruno, QC, Canada).

### DNA manipulation and plasmid construction

Recommendations from the manufacturers of kits were followed for chromosomal DNA isolation (Sigma), plasmid DNA isolation (Qiagen, ON, Canada), extraction of DNA fragments from agarose gels (Qiagen) and purification of PCR products and digested DNA fragments (Qiagen). The exception was the use of supplemental lysostaphin (Sigma) at 200 ug/ml to achieve efficient lysis of *S. aureus* cells. The oligonucleotides (Sigma) used as primers for cloning and plasmid construction are listed in Table 2. Primers were designed to add restriction sites upstream and downstream of the amplified products. PCRs were performed using the Vent DNA polymerase (NEB, Pickering, ON, Canada) or *PfuUltra* high-fidelity DNA polymerase (Agilent Technologies, Mississauga, ON) and cycling times and temperatures were optimized for each primer pair. Plasmid constructs were generated using *E. coli* DH5α (Invitrogen, Burlington, ON, Canada), restriction enzymes (NEB) and the T4 DNA ligase (NEB). Plasmid constructs were validated by restriction digestion patterns and DNA sequencing before electroporation in *S. aureus* RN4220 and in final host strains. Plasmids used in this study are listed in Table 1.

**Table 2:** Primers used in this study.

Primer	Sequence
**Plasmid constructions**	
pBT2-Δ*fnbA*::*ermA*	
*fnbA*_KO_A-FWD	ATCTTGTCTTGTCCCATCCCAAC
*fnbA*_KO_A-REV	TTGCTAGCGATTGTTGCAGCTGTGCTAC
*fnbA*_KO_B-FWD	TTGAATTCCGCTCCCAATTTGTGTTTTC
*fnbA*_KO_B-REV	TTGGATCCAAGGTTAAAGCAGTGGCACC
pFM2	
*sigB*-FWD	TTTGAATTCCATTTAAAACGAATGATTAGGTCA
*sigB*-REV	TTTGGATCCGAGCAGGTGCGAAATAATGG
pFM3	
*fnbA*-FWD	ATATATGTCGACAAAGGGAGATATTATAGTGAAAAACAA
*fnbA*-REV	ATATATGGATCCAACAAATGAAGCAATCAGAAAACA
pFM4	
RNAIII	ATATATCTGCAGAGATCACAGAGATGTGATGGAAA
RNAIII	ATATATGGATCCTGAGGATTAACTCATCCCTTCTT
**qPCR**	
*asp23*-RT-FWD	TCGCTGCACGTGAAGTTAAA
*asp23*-RT-REV	CAGCAGCTTGTTTTTCACCA
*aur*-RT-FWD	ACACAAGAGACGGCGAACTT
*aur*-RT-REV	CTCCCTCTTTTCCTGGTGTG
*fnbA*268-RT-FWD	ACAAGTTGAAGTGGCACAGCC
*fnbA*341-RT-REV	CCGCTACATCTGCTGATCTTGTC
*gyrB*-RT-FWD	GGTGCTGGGCAAATACAAGT
*gyrB*-RT-REV	TCCCACACTAAATGGTGCAA
*hla*-RT-FWD	AATGAATCCTGTCGCTAATGCCGC
*hla*-RT-REV	CTGAAGGCCAGGCTAAACCACTTT
*icaC*-RT-FWD	TTGCGTTAGCAAATGGAGAC
*icaC*-RT-REV	AATGCGTGCAAATACCCAAG
*nuc*1-RT-FWD	CAAGGCTTGGCTAAAGTTGC
*nuc*1-RT-REV	CGTTGTCTTCGCTCCAAATA
*nuc*2-RT-FWD	TCGCTTGCTATGATTGTGGTAGCC
*nuc*2-RT-REV	TACAGGCGTATTCGGTTTCACCGT
RNAIII-RT-FWD	TAATTAAGGAAGGAGTGATTTCAATG
RNAIII-RT-REV	TTTTTAGTGAATTTGTTCACTGTGTC
*sspA*-RT-FWD	ACCTCAAAGGCGAAGCTATG
*sspA*-RT-REV	CCCAATGAATGCCAATGACT

### Generation of CF07-SΔfnbA

An isogenic mutant of the CF07-S strain was constructed, in which the *fnbA* gene was disrupted by the insertion of the *ermA* cassette by homologous recombination. The temperature-sensitive pBT2-Δ*fnbA*::*ermA* plasmid was used combined to a strategy optimized for gene disruption in the SCV CF07-S [31], [54]. The knockout of *fnbA* in strain CF07-SΔ*fnbA* was confirmed by PCR.

### Proteolysis, hemolysis and nuclease activity

Bacterial suspensions (0.5 McFarland standard) prepared for each strain were spotted (2 µl) on BHI agar containing 0.25% glucose or Mueller-Hinton (MH) agar (BD) supplemented with 20 g of non-fat dry milk per liter, MH agar supplement with 5% sheep blood (Oxoid, Nepean, ON, Canada) or DNase test agar (BD). Plates were observed after 24 and 48 h of incubation at 35°C. The three main hemolysins of *S. aureus* (Hla, Hlb and Hld) are active on sheep blood agar plates [38].

### Quantitative real-time PCR (qPCR)

For each qPCR experiment, cultures grown overnight were used to inoculate BHI broth at an A_600 nm_ of 0.1 (100 ml of culture in a 250-ml erlenmeyer). The bacteria were then grown at 35°C with shaking until they reached a specific growth phase. The A_600 nm_ values for the early and the late exponential growth phases were, respectively, 0.4 and 3.0–3.5 for the prototype strains and, 0.25 and 0.9–1.0 for the SCV strains [25]. The stationary phase samples were taken after 12 h of growth. RNA extraction, cDNA synthesis and qPCR were performed as previously described [25], [31], [34] using the primers described in Table 2. The relative expression ratios were calculated by using the cycle threshold (C_t_) of the housekeeping gene *gyrB* (*n*-fold expression = 2^−ΔCt^, where ΔC_t_ represents the difference between the C_t_ of the gene studied and the C_t_ of *gyrB* for each strain).

### Biofilm formation

Strains were incubated on BHI agar or BHI agar 0.25% glucose for 18 h at 35°C. BHI (Figure 1A and 1B) or BHI 0.25% glucose broths were then adjusted to a 0.5 McFarland standard for transfer into wells of a flat-bottom 96-well polystyrene microtiter plate containing a half volume of the same medium and 2× the final concentration of the tested compound (glucose, NaCl, phenylmethylsulfonyl fluoride [PMSF] or CdCl_2_) in a final volume of 200 µl. PMSF (Sigma) was used at a final concentration of 100 µM. For biofilm dispersal assays, two-day old biofilms were incubated for 2 h with proteinase K (Sigma) and DNase I (NEB) at a final concentration of 100 µg/ml and 104 U/ml, respectively. The plates were incubated at 35°C for a total of 48 h and the amount of residual biofilm was revealed as previously described [32], [33].

### Autolysis

Overnight cultures were used to inoculate BHI broths at an A_600 nm_ of 0.1. Bacteria were then grown with shaking at 35°C for 2.5 h (100 ml of culture in a 250-ml erlenmeyer), collected, washed and suspended in a 0.05 M glycine buffer (pH 8.0). This bacterial suspension was mixed 1∶1 with 0.05 M glycine (pH 8.0) supplemented or not with Triton X-100 at a final concentration of 0.05% (initial bacterial suspension to flask volume ratio of 1 to 4). The decrease in A_600 nm_ was monitored as a function of time in order to evaluate bacterial lysis and was expressed relative to the initial turbidity. Bacterial suspensions were maintained at 35°C and agitated throughout the experiment, and were vortexed before each measurement of turbidity. Similar results were also obtained using 0.05 M Tris-HCl (pH 7.2) (data not shown).

### Cell infection assays

Cell infection assays were performed as previously described with few modifications [34]. Briefly, cells were seeded on 12-well Transwell plates (∼1.5×10^5^ cells/insert) and cultured for 9 to 10 days in an air-liquid system. The complete medium in basal compartments was replaced by the invasion medium (1% FBS and no antibiotics) 18 h before assays. Inocula were prepared by suspending bacteria grown for 20 h on BHI agar plates in ice-cold phosphate-buffered saline (PBS). The use of bacteria harvested from agar plates allowed to drastically decrease the occurrence of normal-growing revertants in inocula [34]. Inocula were then washed three times in ice-cold PBS and suspended in the invasion medium supplemented with 0.5% bovine serum albumin (BSA) at a density of approximately 4×10^8^ CFU/ml. Cells were washed twice with PBS and 250 µl of the bacterial suspension was apically added to each insert, which yielded a multiplicity of infection of 100∶1. Invasion was allowed for 3 h, and then inserts were emptied and washed three times with PBS. Invasion medium supplemented with 20 µg/ml of lysostaphin or 20 µg/ml of lysostaphin/50 µg/ml of gentamicin (Sigma) was then added to both apical and basal compartments to kill extracellular bacteria and the cells were incubated for the indicated time. When only lysostaphin was added to the invasion medium, cells were washed once again with PBS and the invasion medium supplemented with lysostaphin was replaced at 24 h post-invasion and/or 1 h before cell lysis to ensure that no bacteria survived or replicated outside cells. Results obtained using both protocols were similar. Whenever required, CdCl_2_ was added to the supplemented invasion media. At the end of the incubation time and following three washes with PBS, cells were detached with 100 µl of 0.25% trypsin and lysed for 10 min by the addition of 400 µl of water containing 0.05% Triton X-100. Lysates were serially diluted 10-fold and plated on agar for CFU determination.

### Cytotoxicity assays

Apical cell culture supernatants from cells infected for 48 h (see Cell infection assays protocol) were harvested. Cells and bacteria were removed from supernatants by two subsequent 5 min centrifugations at 300 and 6800× g, respectively. Samples were stored at 4°C for a maximum of 2 days before use and host cell lysis was evaluated based on the amount of lactate deshydrogenase (LDH) activity as recommended by the manufacturer (Roche, Laval, Qc, Canada).

### Mouse model of pulmonary infection

Colonies grown on BHI agar plates for 20 h were used to prepare bacterial suspensions in cold phosphate-buffered saline (PBS, Sigma). Bacterial suspensions were washed twice and suspended in cold PBS to the number of CFUs required for infection (1×10^8^ CFUs). The test bacteria (CF07-L, CF07-S and CF07-SΔ*sigB*) were used alone or combined (CF07-L with CF07-S or CF07-L with CF07-SΔ*sigB*). For a given experiment, the quantity of bacteria in the mixed inoculum was equivalent to the sum of each inoculum prepared for individual strains. CD-1 female mice (22–24 g, Charles River Canada) were inoculated by intratracheal instillation of 50 µl of the bacterial inoculum using a sterilized 250-µl glass syringe (Hamilton Company) to which was attached a slightly bent 24G feeding needle (Fine Science Tools, Canada). Then, using an otoscope equipped with a speculum (model 21700, Welch Allyn), the larynx was located and the feeding needle was inserted into the trachea for more or less one centimeter. While still looking through the otoscope to maintain the needle in position, the inoculum was slowly instilled. After 48 h of infection, both lungs were harvested and homogenized (Kinematica Polytron homogenizer) in 2 ml of PBS. The CFU content was evaluated by plating logarithmic dilutions of homogenates on TSA and enumerating normal and SCV colonies. The number of SCV colonies was confirmed on TSA containing 2 µg/ml of gentamicin. At least two independent experimental infections were carried out and data were combined.

## Supporting Information

Figure S1 **Expression ratio of the** ***aur*** **gene as a function of growth for strains CF07-L and CF07-S.** QPCR results are expressed according to CF07-L in the early exponential phase of growth. No statistically significant difference was revealed for each growth phase (unpaired *t* test, *n* = 4–5). Results are expressed as means with standard deviations.
(TIF)

Figure S2 **Expression ratio of the** ***sspA*** **gene as a function of growth for strains CF07-S and CF07-SΔ** ***sigB*** **.** QPCR results are expressed according to CF07-S in the early exponential phase of growth. A statistically significant difference between both strains was revealed for the stationary growth phase (**, *P*<0.01; unpaired *t* test, *n* = 3–5). Results are expressed as means with standard deviations.
(TIF)

Figure S3 **Effect of PMSF on the biofilm formation of CF07-SΔ** ***sigB*** **.** Relative biofilm formation of CF07-SΔ*sigB* in the presence of the serine-protease inhibitor PMSF following 48 h of incubation. Results are normalized according to the unexposed condition. No statistically significant difference was revealed (ANOVA with Dunnett's posttest, *n* = 3). Results are expressed as means with standard deviations.
(TIF)

Figure S4 **Effect of menadione on the proteolytic activity of CF07-L, CF07-S and CF07-SΔ** ***sigB*** **.** BHIg milk-agar plates supplemented with 3 µg/ml of menadione were incubated for 48 h at 35°C.
(TIF)

Figure S5 **Effect of Triton-X100 on the autolysis rate of strains CF07-L, CF07-S and CF07-SΔ** ***sigB*** **.** Autolysis of strains CF07-L (A), CF07-S (B) and CF07-SΔ*sigB* (C) as a function of time exposed or not to 0.05% Triton-X100. Results are expressed as percentages of the initial turbidity for each condition. Results are expressed as means with standard deviations (*n* = 3–4).
(TIF)

Figure S6 **Infection of shCFTR Calu-3 cells with strains 8325-4 and DU5883 (** ***fnbAB*** **mutant).** CFU/insert recovered 3 h post-invasion are shown and revealed a statistically significant difference (unpaired *t* test, *n* = 3). Results are expressed as means with standards.
(TIF)

Figure S7 **Infection of Calu-3 and shCFTR_ALTER_ cells with strains CF07-L, CF07-S and CF07-SΔ** ***sigB*** **.** CFU/insert recovered from Calu-3 (A) and shCFTR_ALTER_ cells (B), both expressing a normal CFTR, infected with strains CF07-L, CF07-S and CF07-SΔ*sigB* 48 h post-invasion. Statistically significant differences are indicated (***, *P*<0.001; ANOVA with Tuckey's posttest, *n* = 4–5). Results are expressed as means with standard deviations.
(TIF)

## References

[1] Talbot GH, Bradley J, Edwards JE, Gilbert D, Scheld M, et al (2006) Bad bugs need drugs: an update on the development pipeline from the Antimicrobial Availability Task Force of the Infectious Diseases Society of America. Clin Infect Dis 42: 657–668. 16447111 10.1086/499819

[2] Chambers HF, Deleo FR (2009) Waves of resistance: *Staphylococcus aureus* in the antibiotic era. Nat Rev Microbiol 7: 629–641. 19680247 10.1038/nrmicro2200 PMC2871281

[3] Novick RP (2003) Autoinduction and signal transduction in the regulation of staphylococcal virulence. Mol Microbiol 48: 1429–1449. 12791129 10.1046/j.1365-2958.2003.03526.x

[4] Novick RP, Geisinger E (2008) Quorum sensing in staphylococci. Annu Rev Genet 42: 541–564. 18713030 10.1146/annurev.genet.42.110807.091640

[5] Queck SY, Jameson-Lee M, Villaruz AE, Bach TH, Khan BA, et al (2008) RNAIII-independent target gene control by the *agr* quorum-sensing system: insight into the evolution of virulence regulation in *Staphylococcus aureus* . Mol Cell 32: 150–158. 18851841 10.1016/j.molcel.2008.08.005 PMC2575650

[6] Goerke C, Wolz C (2004) Regulatory and genomic plasticity of *Staphylococcus aureus* during persistent colonization and infection. Int J Med Microbiol 294: 195–202. 15493830 10.1016/j.ijmm.2004.06.013

[7] Senn MM, Giachino P, Homerova D, Steinhuber A, Strassner J, et al (2005) Molecular analysis and organization of the σ^B^ operon in *Staphylococcus aureus* . J Bacteriol 187: 8006–8019. 16291674 10.1128/JB.187.23.8006-8019.2005 PMC1291286

[8] Bischoff M, Dunman P, Kormanec J, Macapagal D, Murphy E, et al (2004) Microarray-based analysis of the *Staphylococcus aureus* σ^B^ regulon. J Bacteriol 186: 4085–4099. 15205410 10.1128/JB.186.13.4085-4099.2004 PMC421609

[9] Bischoff M, Entenza JM, Giachino P (2001) Influence of a functional *sigB* operon on the global regulators *sar* and *agr* in *Staphylococcus aureus* . J Bacteriol 183: 5171–5179. 11489871 10.1128/JB.183.17.5171-5179.2001 PMC95394

[10] Archer NK, Mazaitis MJ, Costerton JW, Leid JG, Powers ME, et al (2011) *Staphylococcus aureus* biofilms: properties, regulation, and roles in human disease. Virulence 2: 445–459. 21921685 10.4161/viru.2.5.17724 PMC3322633

[11] O'Neill E, Pozzi C, Houston P, Smyth D, Humphreys H, et al (2007) Association between methicillin susceptibility and biofilm regulation in *Staphylococcus aureus* isolates from device-related infections. J Clin Microbiol 45: 1379–1388. 17329452 10.1128/JCM.02280-06 PMC1865887

[12] O'Neill E, Pozzi C, Houston P, Humphreys H, Robinson DA, et al (2008) A novel *Staphylococcus aureu*s biofilm phenotype mediated by the fibronectin-binding proteins, FnBPA and FnBPB. J Bacteriol 190: 3835–3850. 18375547 10.1128/JB.00167-08 PMC2395027

[13] Pozzi C, Waters EM, Rudkin JK, Schaeffer CR, Lohan AJ, et al (2012) Methicillin Resistance Alters the Biofilm Phenotype and Attenuates Virulence in *Staphylococcus aureus* Device-Associated Infections. PLoS Pathog 8: e1002626. 22496652 10.1371/journal.ppat.1002626 PMC3320603

[14] Mann EE, Rice KC, Boles BR, Endres JL, Ranjit D, et al (2009) Modulation of eDNA release and degradation affects *Staphylococcus aureus* biofilm maturation. PLoS One 4: e5822. 19513119 10.1371/journal.pone.0005822 PMC2688759

[15] Resch A, Fehrenbacher B, Eisele K, Schaller M, Gotz F (2005) Phage release from biofilm and planktonic *Staphylococcus aureus* cells. FEMS Microbiol Lett 252: 89–96. 16213676 10.1016/j.femsle.2005.08.048

[16] Rice KC, Mann EE, Endres JL, Weiss EC, Cassat JE, et al (2007) The *cidA* murein hydrolase regulator contributes to DNA release and biofilm development in *Staphylococcus aureus* . Proc Natl Acad Sci U S A 104: 8113–8118. 17452642 10.1073/pnas.0610226104 PMC1876580

[17] Boles BR, Horswill AR (2011) Staphylococcal biofilm disassembly. Trends Microbiol 19: 449–455. 21784640 10.1016/j.tim.2011.06.004 PMC3164736

[18] Sendi P, Proctor RA (2009) *Staphylococcus aureus* as an intracellular pathogen: the role of small colony variants. Trends Microbiol 17: 54–58. 19162480 10.1016/j.tim.2008.11.004

[19] Sinha B, Fraunholz M (2010) *Staphylococcus aureus* host cell invasion and post-invasion events. Int J Med Microbiol 300: 170–175. 19781990 10.1016/j.ijmm.2009.08.019

[20] Jarry TM, Memmi G, Cheung AL (2008) The expression of alpha-haemolysin is required for *Staphylococcus aureus* phagosomal escape after internalization in CFT-1 cells. Cell Microbiol 10: 1801–1814. 18466345 10.1111/j.1462-5822.2008.01166.x

[21] Qazi SN, Counil E, Morrissey J, Rees CE, Cockayne A, et al (2001) *agr* expression precedes escape of internalized *Staphylococcus aureus* from the host endosome. Infect Immun 69: 7074–7082. 11598083 10.1128/IAI.69.11.7074-7082.2001 PMC100088

[22] Haslinger-Loffler B, Kahl BC, Grundmeier M, Strangfeld K, Wagner B, et al (2005) Multiple virulence factors are required for *Staphylococcus aureus*-induced apoptosis in endothelial cells. Cell Microbiol 7: 1087–1097. 16008576 10.1111/j.1462-5822.2005.00533.x

[23] Kubica M, Guzik K, Koziel J, Zarebski M, Richter W, et al (2008) A potential new pathway for *Staphylococcus aureus* dissemination: the silent survival of *S. aureus* phagocytosed by human monocyte-derived macrophages. PLoS One 3: e1409. 18183290 10.1371/journal.pone.0001409 PMC2169301

[24] Proctor RA, von Eiff C, Kahl BC, Becker K, McNamara P, et al (2006) Small colony variants: a pathogenic form of bacteria that facilitates persistent and recurrent infections. Nat Rev Microbiol 4: 295–305. 16541137 10.1038/nrmicro1384

[25] Moisan H, Brouillette E, Jacob CL, Langlois-Begin P, Michaud S, et al (2006) Transcription of virulence factors in *Staphylococcus aureus* small-colony variants isolated from cystic fibrosis patients is influenced by SigB. J Bacteriol 188: 64–76. 16352822 10.1128/JB.188.1.64-76.2006 PMC1317593

[26] Brouillette E, Martinez A, Boyll BJ, Allen NE, Malouin F (2004) Persistence of a *Staphylococcus aureus* small-colony variant under antibiotic pressure *in vivo* . FEMS Immunol Med Microbiol 41: 35–41. 15094165 10.1016/j.femsim.2003.12.007

[27] Bates DM, von Eiff C, McNamara PJ, Peters G, Yeaman MR, et al (2003) *Staphylococcus aureus menD* and *hemB* mutants are as infective as the parent strains, but the menadione biosynthetic mutant persists within the kidney. J Infect Dis 187: 1654–1661. 12721946 10.1086/374642

[28] Tuchscherr L, Medina E, Hussain M, Volker W, Heitmann V, et al (2011) *Staphylococcus aureus* phenotype switching: an effective bacterial strategy to escape host immune response and establish a chronic infection. EMBO Mol Med 3: 129–141. 21268281 10.1002/emmm.201000115 PMC3395110

[29] Senn MM, Bischoff M, von Eiff C, Berger-Bachi B (2005) σ^B^ activity in a *Staphylococcus aureus hemB* mutant. J Bacteriol 187: 7397–7406. 16237023 10.1128/JB.187.21.7397-7406.2005 PMC1272976

[30] Kahl BC, Belling G, Reichelt R, Herrmann M, Proctor RA, et al (2003) Thymidine-dependent small-colony variants of *Staphylococcus aureus* exhibit gross morphological and ultrastructural changes consistent with impaired cell separation. J Clin Microbiol 41: 410–413. 12517881 10.1128/JCM.41.1.410-413.2003 PMC149606

[31] Mitchell G, Lamontagne CA, Brouillette E, Grondin G, Talbot BG, et al (2008) *Staphylococcus aureus* SigB activity promotes a strong fibronectin-bacterium interaction which may sustain host tissue colonization by small-colony variants isolated from cystic fibrosis patients. Mol Microbiol 70: 1540–1555. 19007412 10.1111/j.1365-2958.2008.06511.x

[32] Mitchell G, Brouillette E, Seguin DL, Asselin AE, Jacob CL, et al (2010) A role for sigma factor B in the emergence of *Staphylococcus aureus* small-colony variants and elevated biofilm production resulting from an exposure to aminoglycosides. Microb Pathog 48: 18–27. 19825410 10.1016/j.micpath.2009.10.003

[33] Mitchell G, Seguin DL, Asselin AE, Deziel E, Cantin AM, et al (2010) *Staphylococcus aureus* sigma B-dependent emergence of small-colony variants and biofilm production following exposure to *Pseudomonas aeruginosa* 4-hydroxy-2-heptylquinoline-N-oxide. BMC Microbiol 10: 33. 20113519 10.1186/1471-2180-10-33 PMC2824698

[34] Mitchell G, Grondin G, Bilodeau G, Cantin AM, Malouin F (2011) Infection of polarized airway epithelial cells by normal and small-colony variant strains of *Staphylococcus aureus* is increased in cells with abnormal cystic fibrosis transmembrane conductance regulator function and is influenced by NF-κB. Infect Immun 79: 3541–3551. 21708986 10.1128/IAI.00078-11 PMC3165485

[35] Giachino P, Engelmann S, Bischoff M (2001) σ^B^ activity depends on RsbU in *Staphylococcus aureus* . J Bacteriol 183: 1843–1852. 11222581 10.1128/JB.183.6.1843-1852.2001 PMC95078

[36] Marti M, Trotonda MP, Tormo-Mas MA, Vergara-Irigaray M, Cheung AL, et al (2010) Extracellular proteases inhibit protein-dependent biofilm formation in *Staphylococcus aureus* . Microbes Infect 12: 55–64. 19883788 10.1016/j.micinf.2009.10.005

[37] Lauderdale KJ, Boles BR, Cheung AL, Horswill AR (2009) Interconnections between Sigma B, *agr*, and proteolytic activity in *Staphylococcus aureus* biofilm maturation. Infect Immun 77: 1623–1635. 19188357 10.1128/IAI.01036-08 PMC2663138

[38] Herbert S, Ziebandt AK, Ohlsen K, Schafer T, Hecker M, et al (2010) Repair of global regulators in *Staphylococcus aureus* 8325 and comparative analysis with other clinical isolates. Infect Immun 78: 2877–2889. 20212089 10.1128/IAI.00088-10 PMC2876537

[39] Wright JS 3rd, Traber KE, Corrigan R, Benson SA, Musser JM, et al (2005) The *agr* radiation: an early event in the evolution of staphylococci. J Bacteriol 187: 5585–5594. 16077103 10.1128/JB.187.16.5585-5594.2005 PMC1196086

[40] Kiedrowski MR, Kavanaugh JS, Malone CL, Mootz JM, Voyich JM, et al (2011) Nuclease modulates biofilm formation in community-associated methicillin-resistant *Staphylococcus aureus* . PLoS One 6: e26714. 22096493 10.1371/journal.pone.0026714 PMC3214024

[41] Hoiby N, Bjarnsholt T, Givskov M, Molin S, Ciofu O (2010) Antibiotic resistance of bacterial biofilms. Int J Antimicrob Agents 35: 322–332. 20149602 10.1016/j.ijantimicag.2009.12.011

[42] Costerton JW, Stewart PS, Greenberg EP (1999) Bacterial biofilms: a common cause of persistent infections. Science 284: 1318–1322. 10334980 10.1126/science.284.5418.1318

[43] Karlsson A, Saravia-Otten P, Tegmark K, Morfeldt E, Arvidson S (2001) Decreased amounts of cell wall-associated protein A and fibronectin-binding proteins in *Staphylococcus aureus sarA* mutants due to up-regulation of extracellular proteases. Infect Immun 69: 4742–4748. 11447146 10.1128/IAI.69.8.4742-4748.2001 PMC98560

[44] Singh R, Ray P, Das A, Sharma M (2010) Enhanced production of exopolysaccharide matrix and biofilm by a menadione-auxotrophic *Staphylococcus aureus* small-colony variant. J Med Microbiol 59: 521–527. 20110391 10.1099/jmm.0.017046-0

[45] Al Laham N, Rohde H, Sander G, Fischer A, Hussain M, et al (2007) Augmented expression of polysaccharide intercellular adhesin in a defined *Staphylococcus epidermidis* mutant with the small-colony-variant phenotype. J Bacteriol 189: 4494–4501. 17449620 10.1128/JB.00160-07 PMC1913365

[46] Houston P, Rowe SE, Pozzi C, Waters EM, O'Gara JP (2011) Essential role for the major autolysin in the fibronectin-binding protein-mediated *Staphylococcus aureus* biofilm phenotype. Infect Immun 79: 1153–1165. 21189325 10.1128/IAI.00364-10 PMC3067512

[47] Rice KC, Patton T, Yang SJ, Dumoulin A, Bischoff M, et al (2004) Transcription of the *Staphylococcus aureus cid* and *lrg* murein hydrolase regulators is affected by sigma factor B. J Bacteriol 186: 3029–3037. 15126464 10.1128/JB.186.10.3029-3037.2004 PMC400629

[48] Patton TG, Yang SJ, Bayles KW (2006) The role of proton motive force in expression of the *Staphylococcus aureus cid* and *lrg* operons. Mol Microbiol 59: 1395–1404. 16468984 10.1111/j.1365-2958.2006.05034.x

[49] Schulthess B, Bloes DA, Francois P, Girard M, Schrenzel J, et al (2011) The σ^B^-dependent *yabJ-spoVG* operon is involved in the regulation of extracellular nuclease, lipase, and protease expression in *Staphylococcus aureus* . J Bacteriol 193: 4954–4962. 21725011 10.1128/JB.05362-11 PMC3165683

[50] Cui L, Neoh HM, Iwamoto A, Hiramatsu K (2012) Coordinated phenotype switching with large-scale chromosome flip-flop inversion observed in bacteria. Proc Natl Acad Sci U S A 109: E1647–1656. 22645353 10.1073/pnas.1204307109 PMC3382547

[51] Palmer ME, Chaturongakul S, Wiedmann M, Boor KJ (2011) The *Listeria monocytogenes* σ^B^ regulon and its virulence-associated functions are inhibited by a small molecule. MBio 2. 10.1128/mBio.00241-11 PMC3225968 22128349

[52] Charpentier E, Anton AI, Barry P, Alfonso B, Fang Y, et al (2004) Novel cassette-based shuttle vector system for gram-positive bacteria. Appl Environ Microbiol 70: 6076–6085. 15466553 10.1128/AEM.70.10.6076-6085.2004 PMC522135

[53] Palmer ML, Lee SY, Carlson D, Fahrenkrug S, O'Grady SM (2006) Stable knockdown of CFTR establishes a role for the channel in P2Y receptor-stimulated anion secretion. J Cell Physiol 206: 759–770. 16245306 10.1002/jcp.20519

[54] Bruckner R (1997) Gene replacement in *Staphylococcus carnosus* and *Staphylococcus xylosus* . FEMS Microbiol Lett 151: 1–8. 9198277 10.1111/j.1574-6968.1997.tb10387.x

