# Supplementary figures and images for: SigB Is a Dominant Regulator of Virulence in Staphylococcus aureus Small-Colony Variants
Source: PLoS One. 2013 May 21;8(5):e65018. doi: 10.1371/journal.pone.0065018 (PMC3660380; doi:10.1371/journal.pone.0065018)

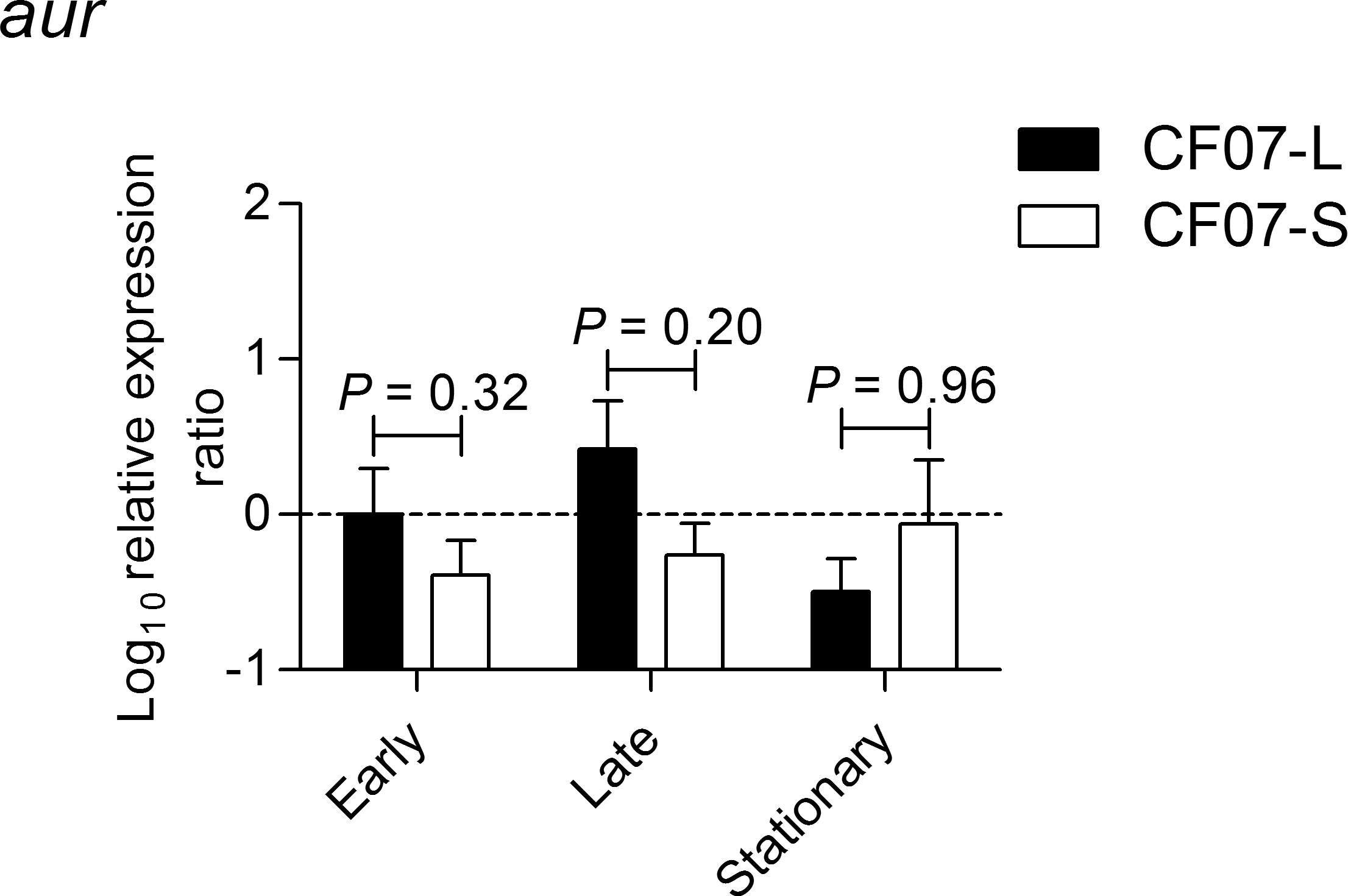

Supplement: Figure S1 — Expression ratio of the aur gene as a function of growth for strains CF07-L and CF07-S. QPCR results are expressed according to CF07-L in the early exponential phase of growth. No statistically significant difference was revealed for each growth phase (unpaired t test, n = 4–5). Results are expressed as means with standard deviations. (TIF) [file pone.0065018.s001.tif]

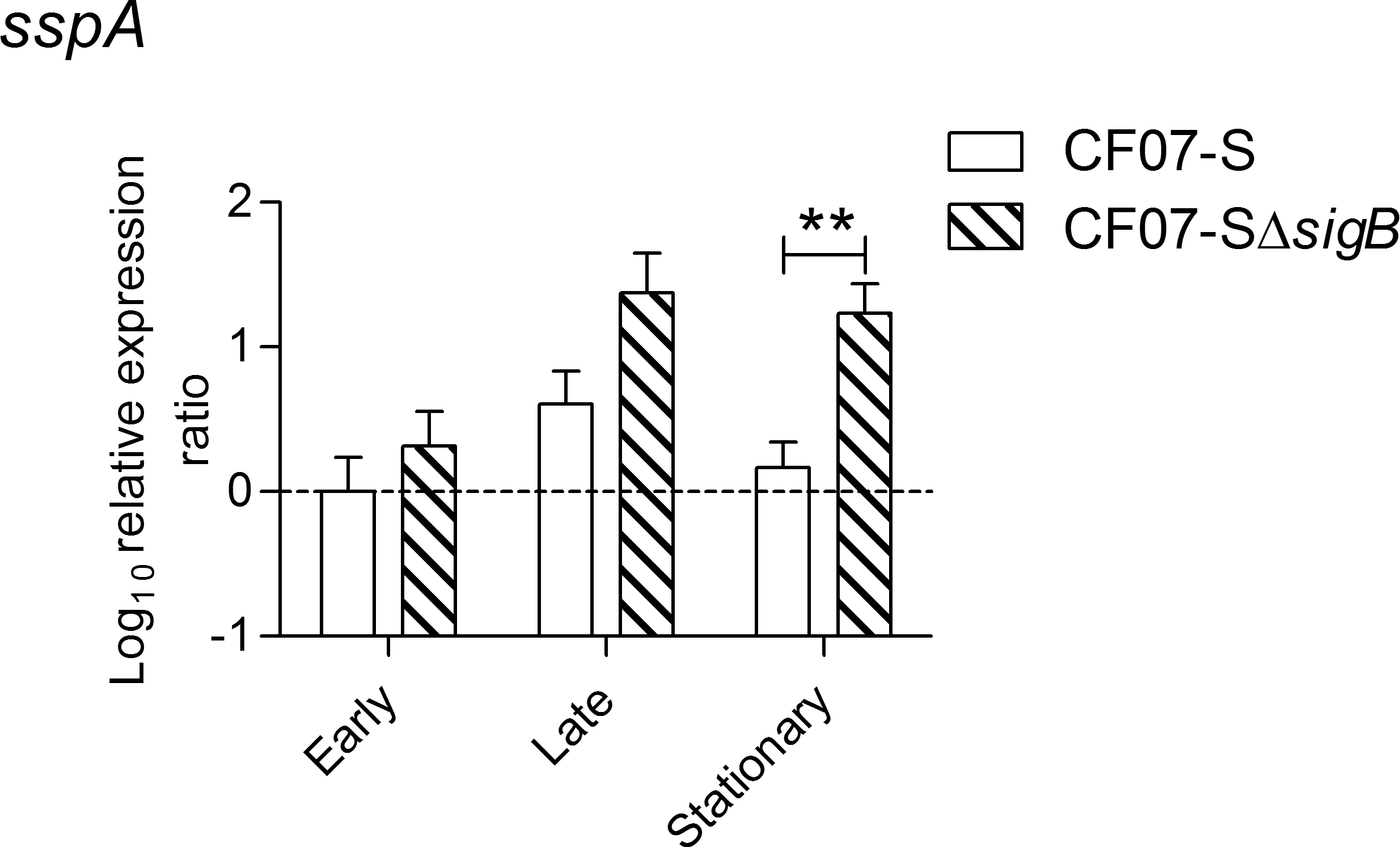

Supplement: Figure S2 — Expression ratio of the sspA gene as a function of growth for strains CF07-S and CF07-SΔ sigB . QPCR results are expressed according to CF07-S in the early exponential phase of growth. A statistically significant difference between both strains was revealed for the stationary growth phase (**, P<0.01; unpaired t test, n = 3–5). Results are expressed as means with standard deviations. (TIF) [file pone.0065018.s002.tif]

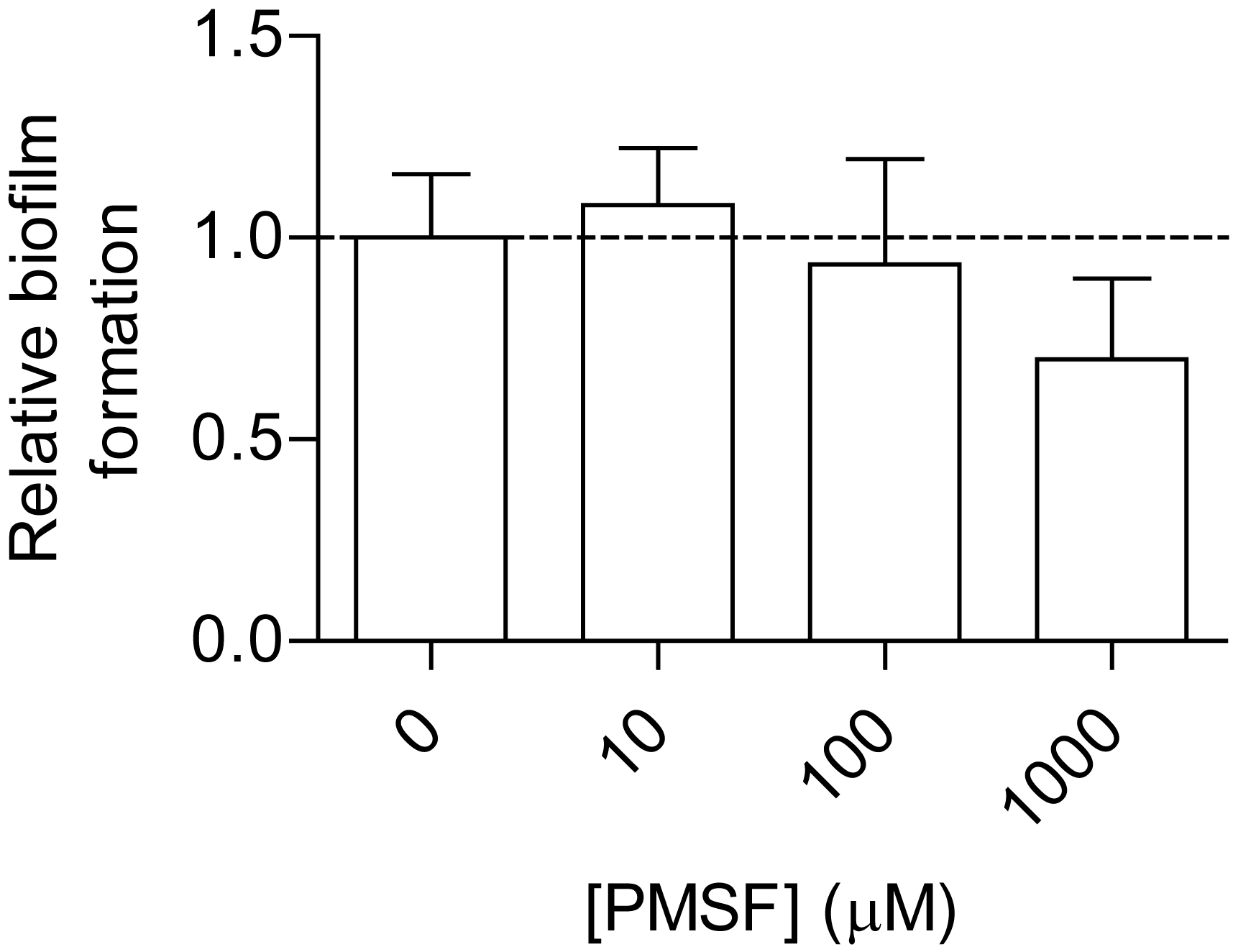

Supplement: Figure S3 — Effect of PMSF on the biofilm formation of CF07-SΔ sigB . Relative biofilm formation of CF07-SΔsigB in the presence of the serine-protease inhibitor PMSF following 48 h of incubation. Results are normalized according to the unexposed condition. No statistically significant difference was revealed (ANOVA with Dunnett's posttest, n = 3). Results are expressed as means with standard deviations. (TIF) [file pone.0065018.s003.tif]

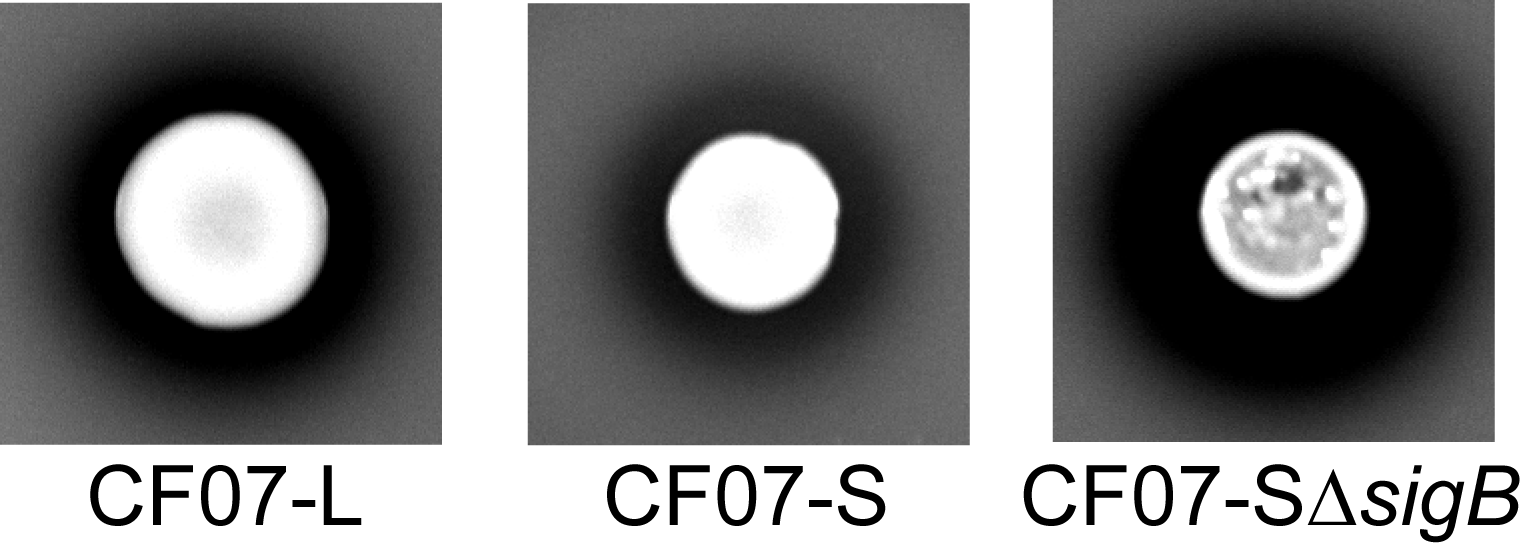

Supplement: Figure S4 — Effect of menadione on the proteolytic activity of CF07-L, CF07-S and CF07-SΔ sigB . BHIg milk-agar plates supplemented with 3 µg/ml of menadione were incubated for 48 h at 35°C. (TIF) [file pone.0065018.s004.tif]

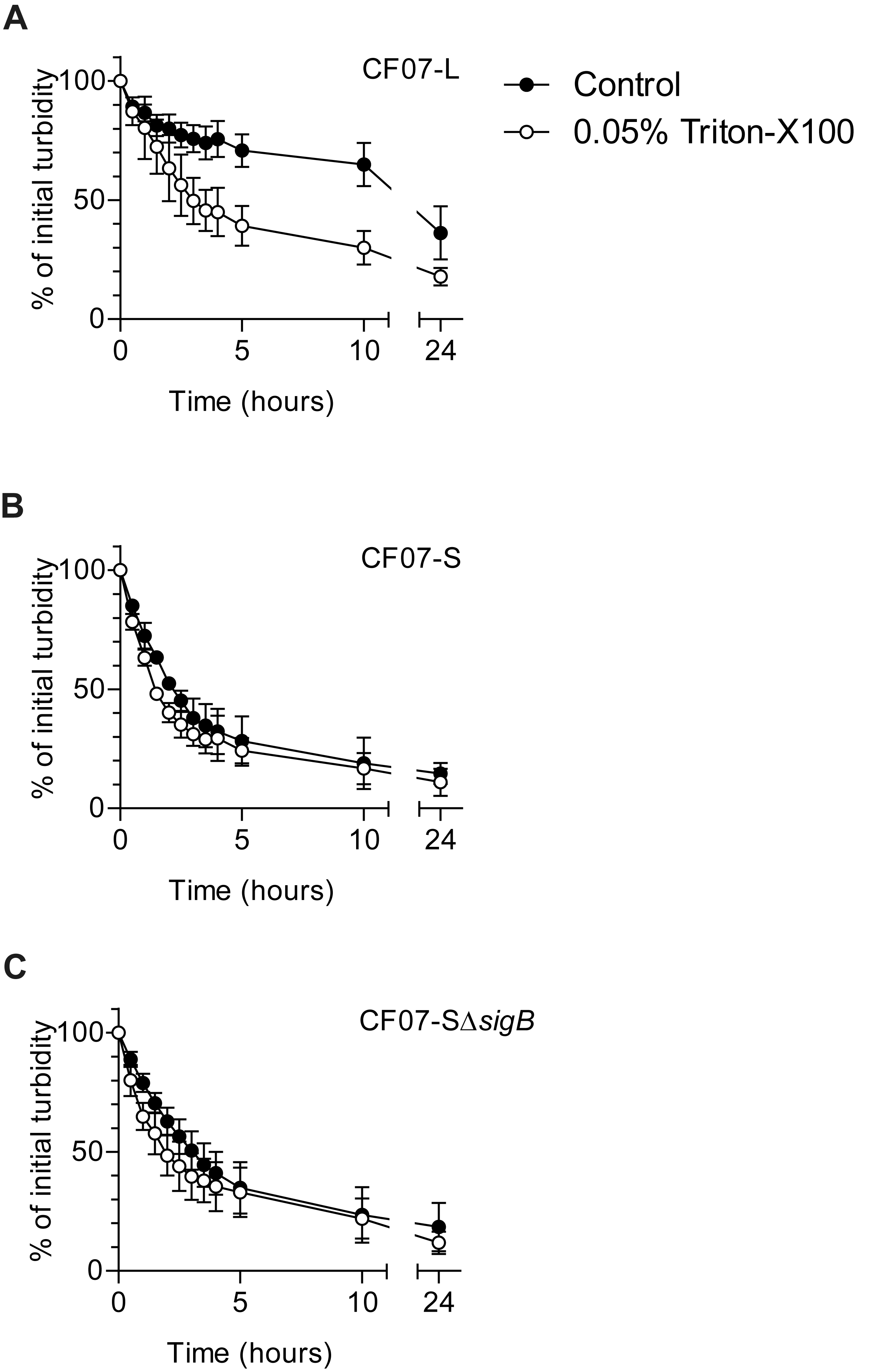

Supplement: Figure S5 — Effect of Triton-X100 on the autolysis rate of strains CF07-L, CF07-S and CF07-SΔ sigB . Autolysis of strains CF07-L (A), CF07-S (B) and CF07-SΔsigB (C) as a function of time exposed or not to 0.05% Triton-X100. Results are expressed as percentages of the initial turbidity for each condition. Results are expressed as means with standard deviations (n = 3–4). (TIF) [file pone.0065018.s005.tif]

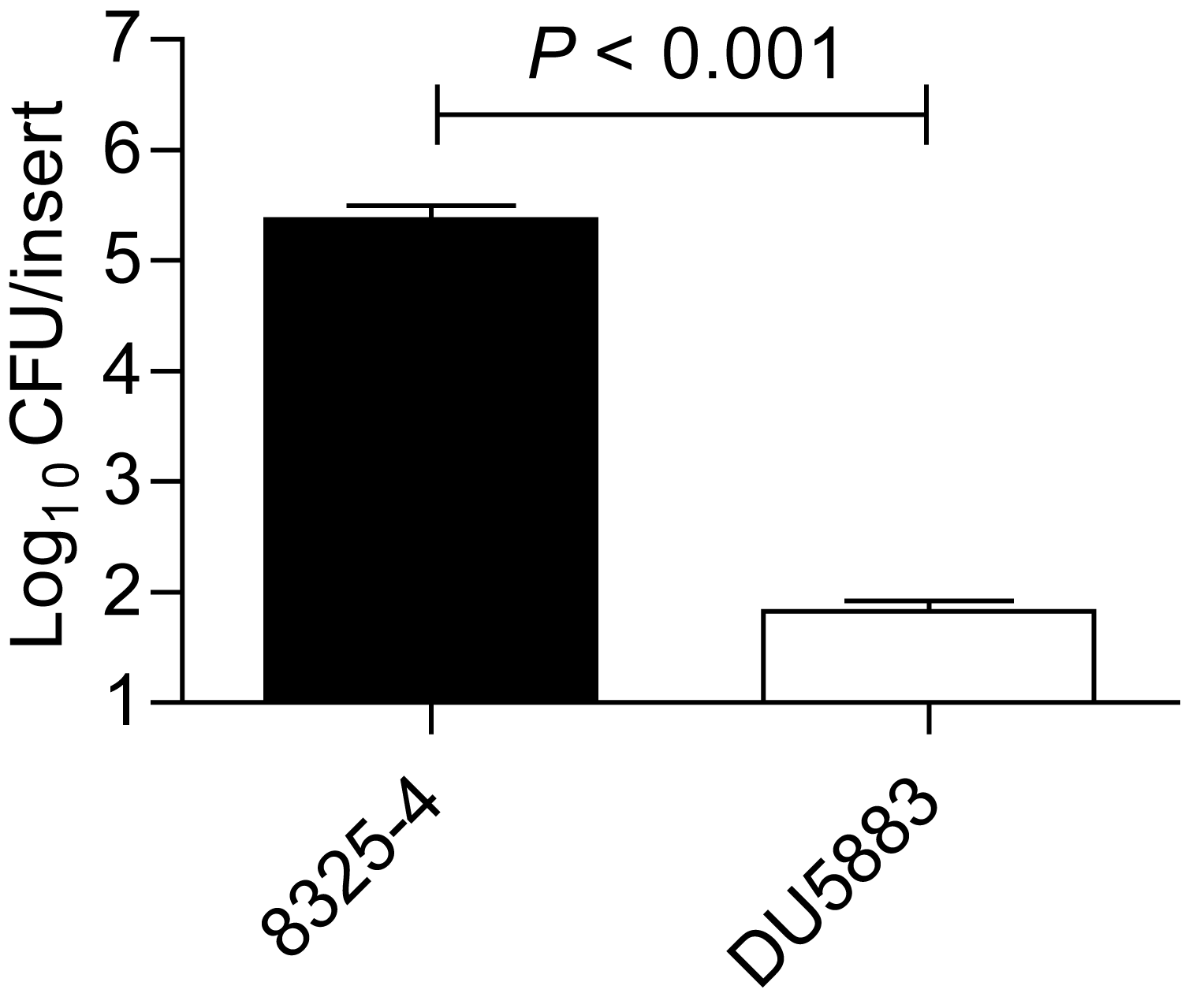

Supplement: Figure S6 — Infection of shCFTR Calu-3 cells with strains 8325-4 and DU5883 ( fnbAB mutant). CFU/insert recovered 3 h post-invasion are shown and revealed a statistically significant difference (unpaired t test, n = 3). Results are expressed as means with standards. (TIF) [file pone.0065018.s006.tif]

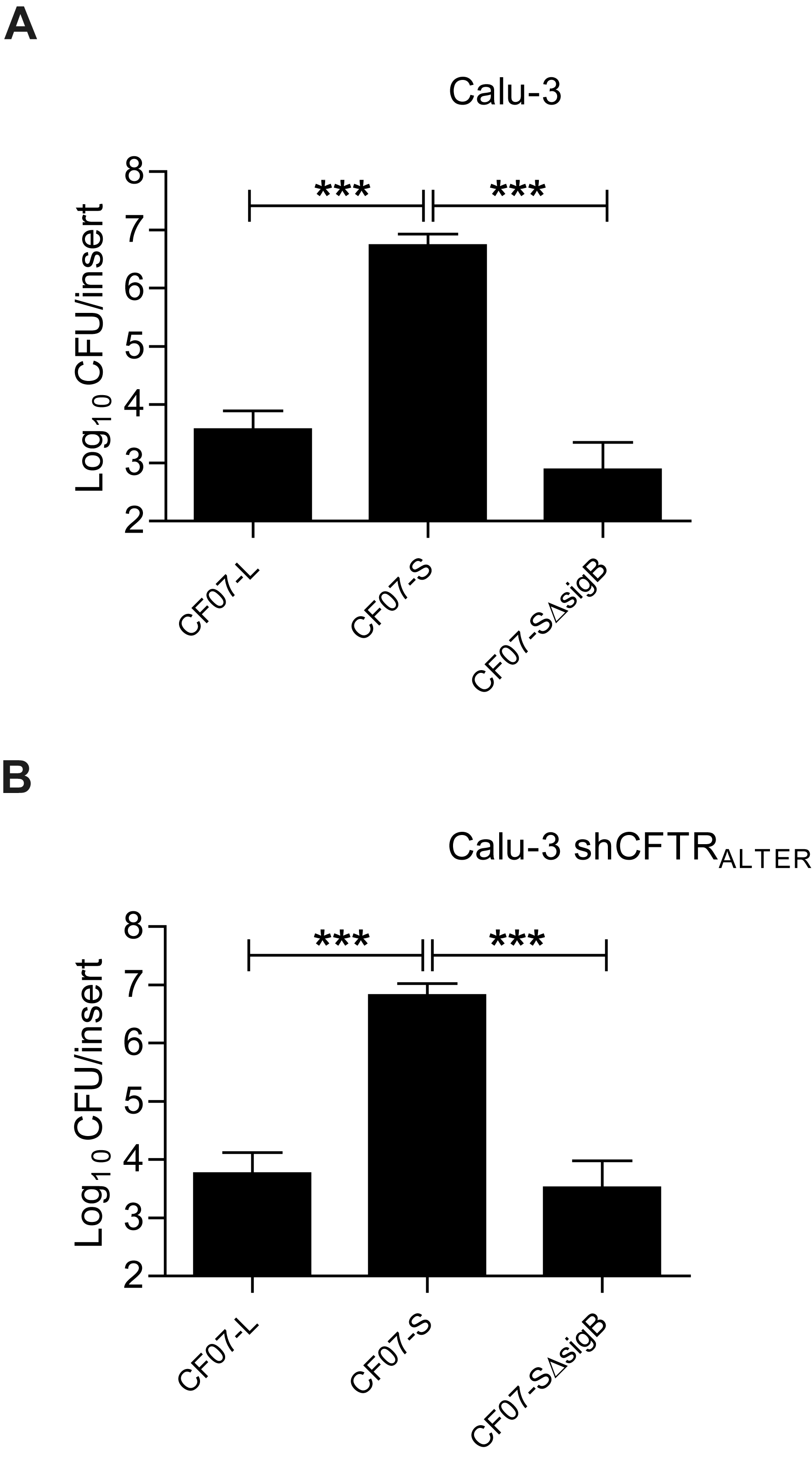

Supplement: Figure S7 — Infection of Calu-3 and shCFTRALTER cells with strains CF07-L, CF07-S and CF07-SΔ sigB . CFU/insert recovered from Calu-3 (A) and shCFTRALTER cells (B), both expressing a normal CFTR, infected with strains CF07-L, CF07-S and CF07-SΔsigB 48 h post-invasion. Statistically significant differences are indicated (***, P<0.001; ANOVA with Tuckey's posttest, n = 4–5). Results are expressed as means with standard deviations. (TIF) [file pone.0065018.s007.tif]
